# Targeting glial fibrillary acidic protein in glaucoma: a monoclonal antibody approach to modulate glial reactivity and neuroinflammation for neuroprotection

**DOI:** 10.1186/s12974-025-03482-8

**Published:** 2025-06-17

**Authors:** Chaoqiang Guan, Linglin Zhang, Kristian Nzogang Fomo, Jie Yang, Norbert Pfeiffer, Franz H. Grus

**Affiliations:** Department of Ophthalmology, University Medical Center of the Johannes Gutenberg-University, Langenbeckstr. 1, 55131 Mainz, Germany

**Keywords:** Glaucoma, Neuroinflammation, Ocular hypertension, Astrocytes, GFAP, Microglia, RGC, Pyroptosis

## Abstract

**Background:**

Glaucoma is a progressive neurodegenerative disorder that leads to irreversible vision loss, with neuroinflammation recognized as a key factor. Overexpression of glial fibrillary acidic protein (GFAP) is linked to glaucoma pathogenesis and plays a pivotal role in astrocyte-driven neuroinflammation. This study aimed to assess the neuroprotective effects of a monoclonal antibody (mAb) targeting GFAP in glaucoma and to elucidate the underlying mechanisms.

**Methods:**

An ocular hypertension (OHT) glaucoma model was established in female Sprague Dawley rats using episcleral vein occlusion. Three doses of GFAP mAb (2.5, 25, 50 µg) or vehicle were administered via intravitreal injection. Retinal nerve fiber layer (RNFL) thickness and photopic electroretinogram were monitored longitudinally. Retinal ganglion cell (RGC) survival and glial responses were evaluated with immunostaining. Western blot and microarray analyses were performed to investigate molecular and pathway alterations. Additionally, a cobalt chloride (CoCl_2_)-induced degenerative R28 cell model was used to validate the protective effects of GFAP mAb in vitro. A bioinformatics re-analysis of a public glaucomatous retina protein dataset was conducted using GSEA, GO, and Cytoscape with GENEMANIA.

**Results:**

OHT resulted in a significant loss of RNFL thickness, PhNR amplitude, and RGC survival, all of which were preserved by GFAP mAb treatment. Retinal astrocyte reactivity was inhibited by GFAPmAb in a dose-dependent manner by suppressing GFAP protein overexpression. Notably, 25 µg GFAP mAb effectively regulated both astrocyte and microglial reactivity, leading to a substantial attenuation of neuroinflammation. Mechanistically, GFAP mAb inhibited the p38 MAPK and NF-κB pathways and the NLRP3/Caspase-1/GSDMD axis. In vitro, GFAP mAb improved R28 cell viability under CoCl_2_ exposure while reducing cell death via inhibition of pyroptosis. Bioinformatic re-analysis highlighted gliosis as a prominent pathway in the glaucomatous retina and indicated GFAP and Caspase1 as central nodes in the putative mechanism network modulated by GFAP mAb.

**Conclusions:**

This study demonstrates that GFAP mAb inhibits astrogliosis and glial-glial activation, exerting neuroprotection through the inhibition of inflammation and pyroptosis. The findings suggest that targeting GFAP represents a promising immunotherapeutic strategy for glaucoma treatment.

**Graphical Abstract:**

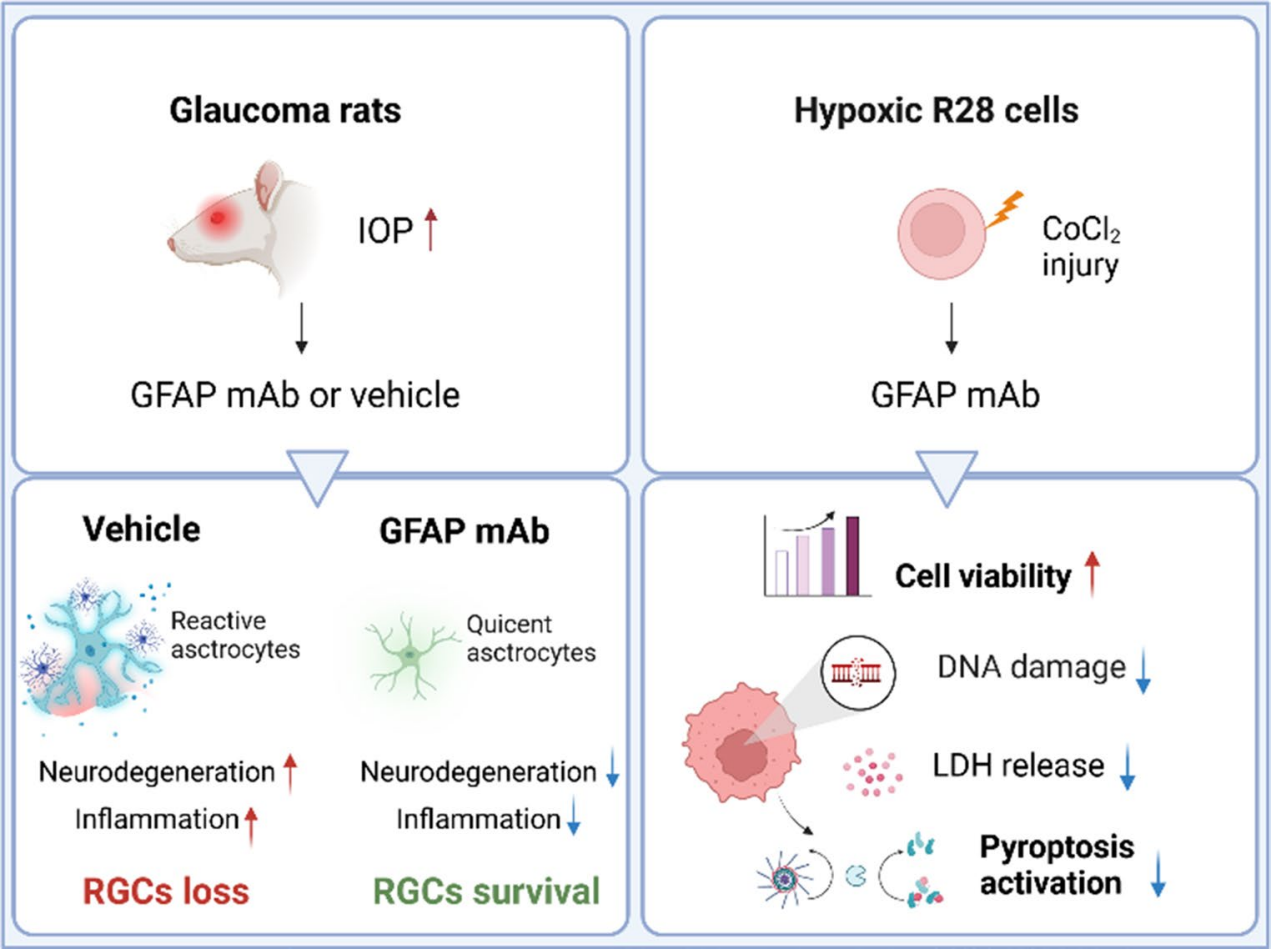

**Supplementary Information:**

The online version contains supplementary material available at 10.1186/s12974-025-03482-8.

## Introduction

Glaucoma, the leading cause of irreversible blindness worldwide [1], is an optic neuropathy characterized by retinal nerve fiber layer (RNFL) thinning, progressive loss of function and death of retinal ganglion cells (RGCs). Elevated intraocular pressure (IOP) is a major risk factor and reducing IOP is currently the only effective clinical strategy [2]. However, glaucoma progression is still observed in a significant number of patients despite successful IOP management [3]. Therefore, therapies that directly promote RGC survival are urgently needed. The underlying mechanisms of glaucomatous neurodegeneration are extremely complex, but abnormal activation of retinal resident glial cells, including astrocytes and microglia, is believed to play a central role [4].

Astrocytes, the most abundant cell type in the central nervous system (CNS) and retina, are critical not only for maintaining neural function but also for contributing to the pathogenesis of neurodegenerative diseases [5]. In the retina, astrocytes are particularly important due to their proximity to and interactions with RGCs in the RNFL [4]. Under normal conditions, they provide neurotrophic, metabolic, and mechanical support and regulate retinal homeostasis against glutamate and reactive oxygen species (ROS) [4]. In response to injury, astrocytes sensitively perceive changes in the microenvironment and switch from a homeostatic state to a reactive state [6], marked by increased proliferation and cellular enlargement [7]. This process is also called “astrogliosis”. Astrocyte reactivity is characterized by the upregulation of glial fibrillary acidic protein (GFAP), a key structural protein in astrocyte intermediate filaments (IF) [8]. Sustained GFAP overexpression induces reactive astrogliosis, driving the release of pro-inflammatory cytokines and activating microglia, thereby amplifying neuroinflammation and contributing to neuronal dysfunction [9, 10]. It has been widely implicated in brain and spinal cord disorders such as Alzheimer’s disease [11], traumatic brain injury [12] and multiple sclerosis [13]. GFAP dysregulation is recognized as an early marker for retinal injury [14], observed in human glaucomatous donor eyes and various experimental glaucoma models [15–17]. GFAP-induced reactive gliosis is implicated in RGC loss following injury [18–20], making GFAP a compelling candidate target for glaucoma treatment.

Targeting GFAP with antibodies represents a promising immunotherapeutic strategy for neutralizing its overexpression. Indeed, GFAP auto-antibodies have been detected in human serum and aqueous humor [21, 22]. Previous studies have shown that natural anti-GFAP autoantibodies are down-regulated in primary open angle glaucoma patients [22], suggesting an impaired protective autoimmunity. However, the exact role of GFAP antibodies in glaucomatous neurodegeneration is to be determined and the mechanism remains largely unknown. To address this issue, we established an experimental chronic ocular hypertension model to investigate whether a recombinant monoclonal anti-GFAP antibody (GFAP mAb) can modulate glial reactivation and promotes the restoration of structural and functional damage. Postmortem analysis focused on astrocyte- and microglia-associated neuroinflammation, with multiple inflammatory mediators and pathways analyzed via a highly sensitive protein microarray. Additionally, an in vitro retinal degeneration model was applied to investigate the underlying mechanisms of GFAP mAb at the intracellular level by examining mitochondrial damage and cell death. Finally, we carried out a bioinformatic re-analysis of a public glaucoma proteomics dataset to explore the putative mechanism of GFAP mAb regulation within the involved protein molecular network.

## Methods

### Ethics statement

All animals were treated in accordance with the institutional guidelines of the Johannes Gutenberg University, Mainz, Germany. All experiments were conducted in accordance with the Association for Research in Vision and Ophthalmology (ARVO) guidelines, and the methods and procedures were reviewed and approved by the National Investigation Office of the State of Rhineland-Palatinate in Koblenz, Germany (License number: G21-1-029).

### Experimental design

**Fig. 1:**
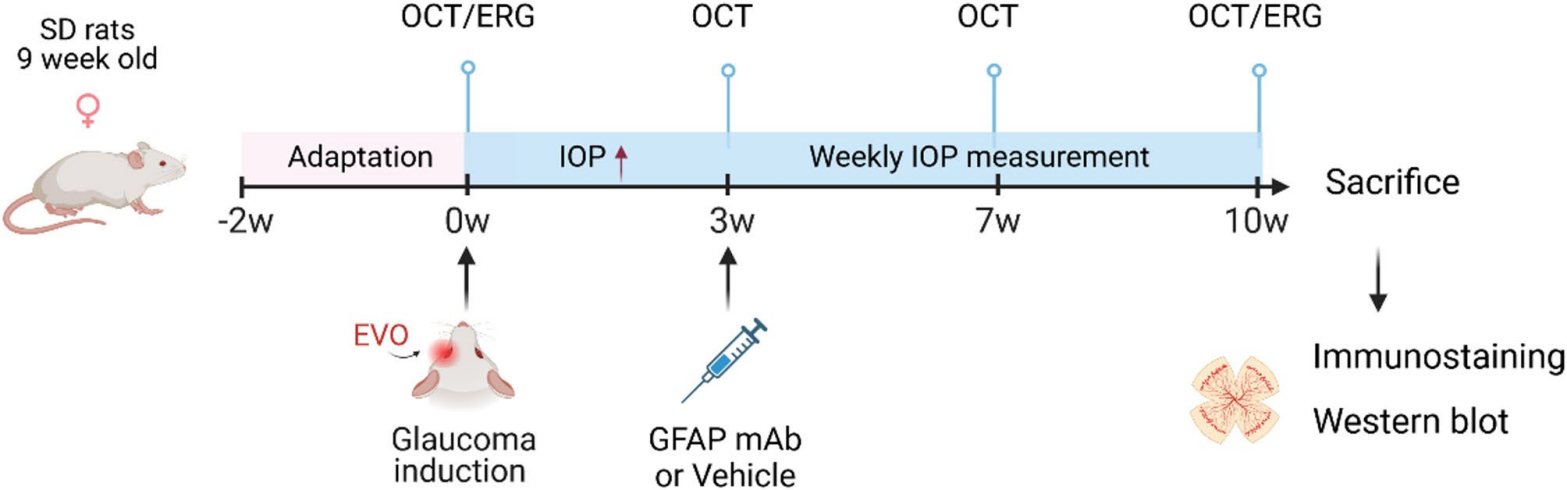
Experimental timeline and procedure overview. This figure illustrates the key steps and timeline of the animal experiment. EVO was performed exclusively on the left eye (OS). Three different dosages (2.5 µg, 25 µg, 50 µg) of GFAP mAb or vehicle (PBS) were administrated via intravitreal injection on the OS (*n* = 7 per group). SD rats: Sprague Dawley rats; OCT: optical coherence tomography; ERG: electroretinogram; IOP: intraocular pressure; EVO: episcleral vein occlusion. (created with BioRender.com)

Healthy female Sprague Dawley (SD) rats (Charles River, Sulzfeld, Germany) were used in this study. The animals were housed in a near pathogen-free environment under standard conditions (21 °C, 12 h/12 h light-dark cycle) with ad libitum access to food and water. After a two-week acclimation period, experiments began when animals were nine weeks old. Each animal underwent baseline pre-measurements including IOP measurements, OCT, and ERG analysis. At week 0, all animals received episcleral vein occlusion (EVO) surgery to induce chronic IOP elevation exclusively in the left eye (OS). Three weeks post-EVO, once IOP had been stably elevated, GFAP mAb (MBS438192, MyBiosource, San Diego, CA, USA) or PBS was administered into the OS by intravitreal injection (IVI). Animals were randomly assigned to one of four treatment groups: 2.5 µg GFAP mAb (*n* = 7), 25 µg GFAP mAb (*n* = 7), 50 µg GFAP mAb (*n* = 7), and vehicle (PBS, *n* = 7). Randomization was performed manually by the investigator administering the injections who was blinded to the animals’ baseline measurements. The right eyes (OD) of the animals remained untreated and served as the normal control. All outcome assessments, including IOP measurement, OCT, ERG and postmortem analyses, were performed by investigators blinded to group allocation. IOP was monitored throughout the experiment. OCT imaging was performed longitudinally at week 3 (w3), week 7 (w7), and week 10 (w10). At the end of the experiment (week 10), ERG was performed, and the animals were sacrificed by carbon dioxide overdose (Fig. 1).

### Establishment of ocular hypertension (OHT) glaucoma animal model

Chronic OHT was induced by EVO surgery. Briefly, the animals were systemically anesthetized with an intramuscular injection of medetomidine (0.185 ml/kg, Zoetis Deutschland GmbH, Berlin, Germany), and oxybuprocaine eyedrops (OmniVision GmbH, Puchheim, Germany) were applied to the OS for topical anesthesia. Under anesthesia, the conjunctiva was incised parallel to the limbus by 1 mm to expose the episcleral veins. Three of the four episcleral veins were isolated, thermally cauterized, and then incised to confirm a complete interruption of blood flow. Then, the conjunctival incision was sutured to its original position. Ofloxacin ointment (Pharma Stulln GmbH, Stulln, Germany) was applied to the cornea to prevent infection. Bepanthene ophthalmic ointment (Bayer Vital GmbH, Leverkusen, Germany) was applied to the contralateral eye to maintain the cornea moist.

### IOP monitoring

IOP was measured weekly between 9 a.m. and 11 a.m. using a TonoLab rebound tonometer (iCare, Espoo, Finland). Measurements were taken with the animals awake and calm. The TonoLab was held so that its probe pointed horizontally at the cornea, briefly touching the surface. Ten consecutive proper measurements were taken and recorded to calculate the average value.

### Intravitreal injection

Under systemic anesthesia, animals were positioned in a surgical holder. After topical anesthesia with oxybuprocaine eye drops, pupils were dilated with tropicamide (Pharma Stulln, Stulln, Germany) to enhance the view of the fundus. Using a 10 µL Hamilton syringe (Sigma Aldrich, Steinheim, Germany) with a 33G needle, 3.5 µL of GFAP mAb or vehicle was injected into the posterior chamber. To prevent reflux, the needle was left in the posterior chamber for 30 s then slowly withdrawn from the eye. Ofloxacin ointment was applied to prevent infection.

### Optical coherence tomography

Optical coherence tomography (OCT) represents a non-invasive method for three-dimensional retina imaging in vivo. We used spectral-domain OCT (SD-OCT, Heidelberg Engineering GmbH, Heidelberg, Germany) to monitor the RNFL thickness longitudinally. Under anesthesia with medetomidine and topical oxybuprocaine, pupils were dilated with tropicamide before OCT examination. The scanning and analysis were conducted as previously described [23]. A 12° diameter circular B-scan around the optic disc was acquired, and the RNFL thickness was analyzed semi-automatically using Heidelberg Eye Explorer software.

### Photopic electroretinogram recording

Photopic Ganzfeld electroretinogram (ERG) recordings were conducted at baseline (week 0) and 10 weeks post-EVO surgery to assess the retinal function. Under systemic and topical anesthesia, pupils were dilated and then rats were positioned on a custom platform with eyes fully exposed as previously described [23]. Four electrodes (Roland Consult, Brandenburg, Germany) were then connected to the rats: two gold ring-electrodes were placed on both corneas with moderate pressure, one needle-electrode was inserted subcutaneously into the neck, and one needle-electrode was applied to the tail. Methocel (OmniVision, Puchheim, Germany) was applied to cover the corneas for moisture and electrode contact. The photopic Ganzfeld ERG was recorded using an RETI system (Roland Consult, Brandenburg, Germany). Rats were light-adapted (40 cd∙s∙m^− 2^) for 20 min. After observation of the appropriate impedance and good baseline signal, the intensities of white stimuli flash used to measure the retina functionality were set to − 0.15, 0.23, 0.61, 0.99, and 1.37 log_10_ cd∙s∙m^− 2^, respectively. The stimuli flash frequency was set to 0.33 Hz, and the plot time for each recording was 512 ms. After each stimulus, 25 responses in total were recorded, and artifacts were excluded automatically by the system. After recording, Ofloxacin ointment was applied to the corneas to prevent infection.

### Immunofluorescence staining in retinal flat mounts

Following euthanasia, rat eyes were enucleated and transferred to PBS. Retinas were carefully dissected and cut into four quarters. Two quarters were snap-frozen in liquid nitrogen and stored at -80 ℃ for protein analysis. The remaining quarters were fixed in 4% paraformaldehyde (PFA, Histofix, Roth, Karlsruhe, Germany) for 30 min, then dehydrated in 30% sucrose overnight. For immunofluorescence staining, retinas were washed with PBS and incubated with a blocking buffer consisting of 10% normal donkey serum (NDS, Sigma-Aldrich, St. Louis, MO, USA) and 0.3% Triton X-100 (Sigma-Aldrich, St. Louis, MO, USA) in PBS for 2 h at room temperature. Primary antibodies, including anti-Brn3a conjugated with Alexa Fluor 594 (1:250, sc-8429 AF594, Santa Cruz, CA, USA), anti-GFAP (1:500, PA3-16727, Invitrogen, CA, USA), anti-Iba1 (1:500, 10904-1-AP, Proteintech, IL, USA), diluted in the blocking solution, were applied overnight at 4℃. After washing, retinas were incubated with corresponding secondary antibodies, including donkey anti-mouse IgG Alexa Fluor 568 antibody (1:400, ab175472, Abcam, Cambridge, UK), donkey anti-rabbit IgG Alexa Fluor 488 antibody (1:400, ab150073, Abcam, Cambridge, UK) for 2 h at room temperature, followed by DAPI (1:1000, Thermo Fisher Scientific, Rockford, IL, USA) staining for nuclear labeling. Slides were mounted with Aqua-Poly/Mount mounting medium (Polysciences, Warrington, PA, USA). Fluorescent images were captured using a fluorescence microscope (BZ-X800, Keyence, Osaka, Japan).

### Image analysis

**Fig. 2:**
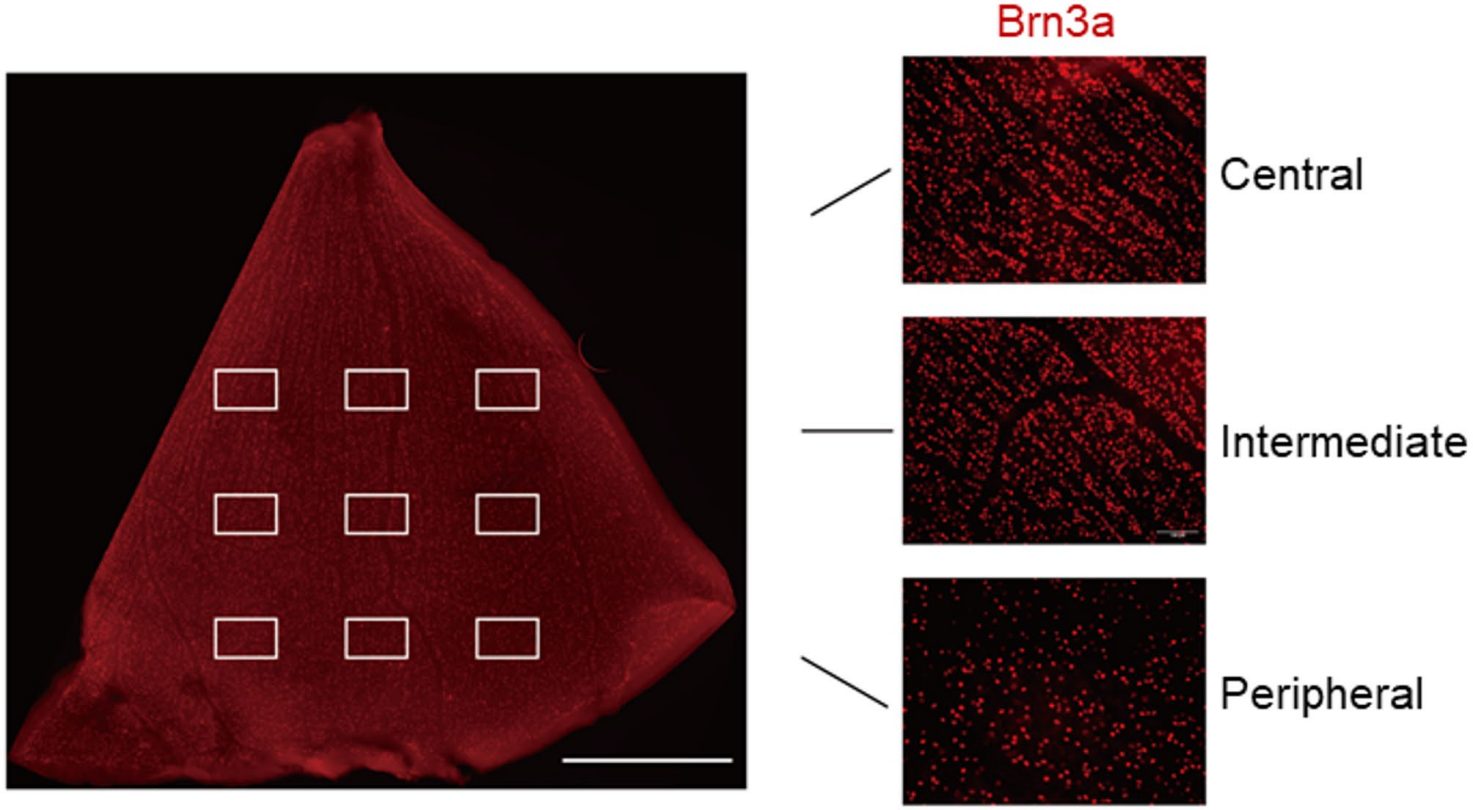
Schematic of the three retinal flat mount regions for RGC density analysis. This is a stitched scan of Brn3a immunostaining across a quarter of the retinal flat mount, generated using the microscope’s internal software (scale bar = 1 mm)

RGC density was assessed by counting Brn3a-positive cells in retinal flat mounts using ImageJ. (version 1.54, NIH, Bethesda, MD, USA). Retinas were divided into central, intermediate, and peripheral zones based on their distance from the optic nerve head, with three images per zone for a total of nine per flat mount (Fig. 2). Astrocyte and microglia reactivity were analyzed by quantifying the GFAP-labeled area and Iba1-positive cell number using ImageJ software as previously described [16, 24, 25]. For each retinal flat mount, three images from the intermediate region were analyzed and averaged. Analysis was performed in a blinded manner by two investigators.

### CoCl_2_-induced R28 cell degeneration model in vitro

The immortalized rat retina cell line, R28 (RRID: CVCL_ 5I35), was a generous gift from Prof. Gail M. Seigel (University of Rochester, USA). The R28 cells were cultured in DMEM with 10% fetal bovine serum, 1% Vitamin C, 1% Non-Essential Amino Acids, and 1% penicillin/streptomycin in T75 flasks. Cells were maintained at 37 °C in a humidified 5% CO_2_ environment. Following exposure to 50 µM CoCl_2_ for 24 h, cells were treated with GFAP mAb at concentrations ranging from 2 µg/ml to 8 µg/ml. The optimal concentrations of GFAP mAb were determined based on the highest cell viability and applied in subsequent experiments.

### Cell viability assay

After treatments, R28 cell viability was assessed by the MTS assay (#G3580; Promega Corporation, Madison, WI, USA) with 20 µL reagent in each well incubated at 37 °C for 1 h. Cells that were not treated with CoCl_2_ or antibodies served as normal controls with 100% cell viability. Cell viability was determined by measuring the absorbance at 450 nm using a microplate reader (Multiskan Ascent, Thermo Labsystems, Massachusetts, USA). Cell viability was calculated using the formula: viability (%) = OD (measured value) − OD (blank value)/OD (control value) − OD (blank value) × 100%.

### TUNEL assay

The TUNEL assay (12156792910, Roche-Applied-Science, Mannheim, Germany) was conducted to assess in situ cell death. Following treatments, cells were fixed and permeabilized with 0.1% Triton X-100 for 2 min on ice, and incubated with 50 µL of TUNEL reaction mixture for 1 h at 37 °C in the dark. DAPI staining was used to visualize the nuclei. Fluorescence microscopy (Eclipse TS 100; Nikon, Tokyo, Japan) was used to visualize TUNEL-positive cells. For each experiment, 10 random images were recorded in each group.

### Mitochondrial membrane potential measurements

The Mitochondrial membrane potential (MMP) was determined using a JC-10 assay kit (786–1541, Gbiosciences, Saint Louis, MO, USA), according to the manufacturer’s manual. Following the experimental procedures, the cells were stained with the JC-10 dye (15 µM) for 40 min at 37 °C in the dark. Stained cells were analyzed using a fluorescence microplate reader (Fluoroskan Ascent FL, Thermo Labsystems, Massachusetts, USA). The green fluorescence was read at Ex/Em = 485/535 nm and the orange fluorescence was read at Ex/Em = 530/590 nm. The MMP was calculated as the ratio of orange to green fluorescence.

### Lactate dehydrogenase (LDH) assay

LDH release was measured to assess cell rupture, using the CyQUANT™ LDH Cytotoxicity Assay (C20300, Invitrogen, Rockford, IL, USA). For the spontaneous LDH activity control, 10 µL of sterile ultrapure water was added to one set of triplicate wells of cells. For maximum LDH activity control, 10 µL of 10X Lysis Buffer was added to the set of triplicate wells followed by incubation at 37 °C for 45 min. After treatment, 50 µL supernatant was sampled from each well to mix with 50 µL of LDH reaction solution and incubated at 37 °C for 30 min. LDH release was measured by the absorbance values at 490 nm. The LDH release rate was calculated using the formula: % LDH release = (experimental groups - spontaneous control)/ (maximum control - spontaneous control) ×100.

### Immunocytochemistry

After treatment, cells were fixed for 15 min and then washed with PBS. Permeabilization and blocking were performed using a blocking buffer consisting of 10% NDS and 0.5% Triton X-100 in PBS for 1 h at room temperature. Cells were then incubated with the primary antibody (anti-GSDMD, #39754, Cell Signaling Technology, Danvers, MA, USA) overnight at 4 °C, followed by incubation with the secondary antibody donkey anti-rabbit IgG Alexa Fluor 488 antibody (1:400, ab150073, Abcam, Cambridge, UK) for 1 h at room temperature. Nuclei were counterstained with DAPI (1:1000), and fluorescence images were acquired using a fluorescence microscope (Eclipse TS 100; Nikon, Tokyo, Japan).

### Western blot analysis

Proteins were extracted from retinal tissues and R28 cells using T-PER buffer (78510, Thermo Fisher Scientific, Rockford, IL, USA) and RIPA buffer (89900, Thermo Fisher Scientific, Rockford, IL, USA) containing a 1% Protease and Phosphatase Inhibitor Cocktail (78440, Thermo Fisher Scientific, Rockford, IL, USA) respectively. Equal amounts of protein (30 ~ 40 µg) per lane were separated on NuPAGE 4–12% Bis-Tris gels (NP0321, Thermo Fisher Scientific, Rockford, IL, USA), followed by transferring to a 0.45 μm PVDF membrane (88518, Thermo Fisher Scientific, Rockford, IL, USA). After blocking, membranes were probed with primary and secondary antibodies (supplementary Table 1) and visualized using SignalFire™ Elite ECL reagent (12757, Cell Signaling Technology, Massachusetts, USA). Visual detection was performed using the Fluor Chem E system (ProteinSimple, San Jose, California, USA). The densitometric analysis was performed using ImageJ and β-actin or β-Tubulin were used as a reference protein to normalize the target proteins.

### High-throughput protein analysis using microarray

Microarray analysis was performed as previously described [26]. Briefly, the microarray was prepared using a noncontact microarray printer (SciFLEXARRAYER S3; Scienion, Berlin, Germany). Antibodies (supplementary Table 2) were spotted in triplicate onto nitrocellulose-coated slides (AVID Oncyte, NC 16 Pad slides; Grace Bio-Labs, Bend, OR, USA). Protein samples were transferred to labeling buffer (0.05 M sodium borate buffer, pH 8.5) and labeled with 1 µL of fluorescent dye (DyLight 650 NHS Ester; Thermo Fisher Scientific, Rockford, IL, USA). Samples were incubated overnight at 4 °C. A negative control using labeled PBS was prepared in the same manner. The labeling reaction was quenched by adding 10 µL of quenching solution (Tris-HCl, pH 8.8) and incubating for 30 min at room temperature in the dark. Unbound dye was removed using Zeba desalting plates (7k MWCO; Thermo Fisher Scientific) according to the manufacturer’s protocol. Slides were mounted in 16-well chambers (ProPlate Multiwell Chambers; Grace Bio-Labs) and blocked with a blocking buffer (Super G; Grace Bio-Labs) for 1 h at 4 °C. After blocking, the slides were washed three times with PBST (PBS with 0.5% Tween 20). Each well was incubated with either the labeled protein sample or dye-labeled PBS overnight at 4 °C under moderate shaking. Slides were then washed twice with PBST and twice with ultrapure water, dried for 1 min, and scanned using a CCD camera-based array reader (SensoSpot; Sensovation, Radolfzell, Germany) with a 200 ms exposure time in the red channel. The resulting array images were saved as 16-bit TIF files for further analysis.

### Microarray data processing

Imagene software (version 5.5; BioDiscovery Inc., Los Angeles, CA, USA) was employed to quantify spot intensities in the microarray. Spots that did not meet the quality control criteria were flagged and manually removed. The median spot intensity was used for further analysis. Local background intensity was subtracted from each spot, and the intensity of the negative control was also subtracted to eliminate nonspecific signals. Finally, the mean intensity of the triplicate spots was calculated and used for subsequent analysis.

### Bioinformatics analysis

We re-analyzed the publicly available retinal protein expression profiling of EVO-induced glaucoma (ID: PXD005258) from the PRIDE database (https://www.ebi.ac.uk/pride/archive/projects/PXD005258; accessed on 10 December 2024) [27]. The bioinformatic analysis was carried out on samples from glaucomatous eyes (7 weeks of elevated IOP) and control eyes. A Student’s two-sided t-test was performed to identify proteins with significant changes between glaucoma and control group, with a *p*-value < 0.05 considered statistically significant. Gene Set Enrichment Analysis (GSEA) was performed to identify classes of proteins that are over-represented in glaucomatous eyes. The top 20 features were selected for Gene Ontology (GO) enrichment analysis. We retrieved the expression profiles of the proteins GFAP, Vim, S100B, HSP90B1, EEF2, and ANXA1, based on their association with astrogliosis from current literature data. A protein-protein interaction network was generated and visualized using Cytoscape 3.10.2 with GENEMANIA.

### Statistical analysis

Statistical analyses were performed using GraphPad Prism 10.0 (La Jolla, CA, USA). The normality of data distribution was assessed using the Shapiro-Wilk test. For comparisons across multiple groups, one-way analysis of variance (ANOVA) was applied, followed by Tukey’s post hoc test for pairwise comparisons. The Brown-Forsythe test was used to evaluate the homogeneity of variances between groups. In cases where significant heterogeneity of variances was detected, the Welch ANOVA test was employed, followed by the Tamhane T2 post hoc test. For analysis of ERG data, interocular differences (OD vs. OS) within each treatment group were assessed using paired Student’s t-tests, followed by Holm-Šidák’s multiple comparisons correction to control for Type I error across groups. Data are presented as mean ± standard deviation (SD), and *p*-value < 0.05 was considered statistically significant.

## Results

### Characterization of IOP elevation and progressive RNFL loss in an EVO-induced glaucoma model

**Fig. 3:**
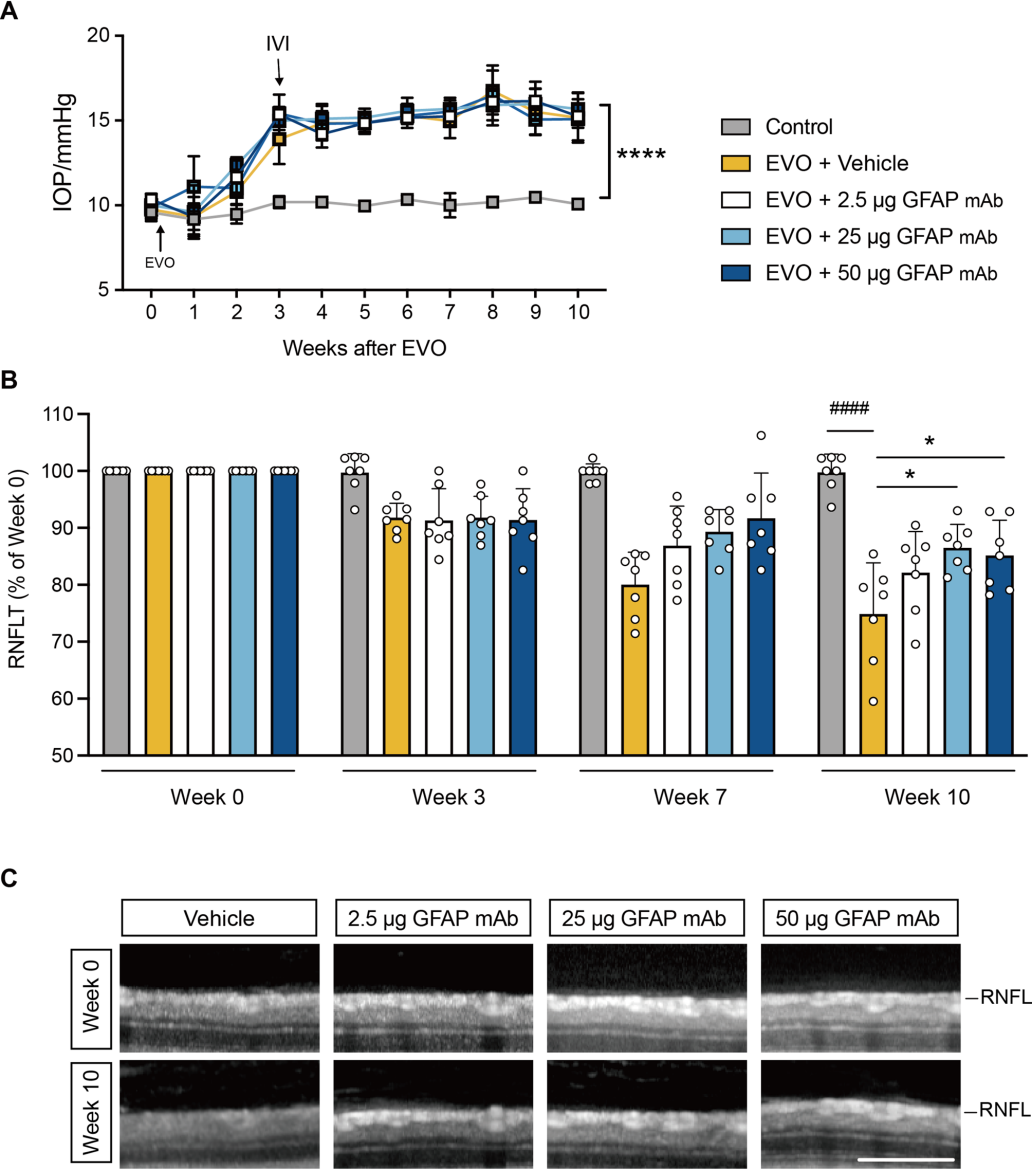
Longitudinal IOP and RNFL thickness monitoring of glaucomatous rat retinas. (**A**) IOPs were monitored weekly using rebound tonometry. Vehicle or three doses of GFAP mAb were administered to the OS at week 3 via intravitreal injection (IVI). The IOP in the OS increased three weeks post-EVO and remained significantly higher than that in the OD by week 10. ****p < 0.0001; one-way ANOVA with Tukey’s post-hoc test. (**B**) Quantification of retinal nerve fiber layer (RNFL) thickness, presented as a percentage of baseline (week 0), shows that GFAP mAb treatment attenuated RNFL loss in a dose-dependent manner. Statistical significance: ^####^*p* < 0.0001 vs. OD control; *p < 0.05 vs. vehicle; one-way ANOVA with Tukey’s post-hoc test. (**C**) Representative optical coherence tomography (OCT) images of each group at week 0 and week 10 (scale bar = 200 μm). Data are presented as the mean ± SD; *n* = 7 per group

Following EVO surgery, the OS of rats developed characteristics of OHT glaucoma, with IOP increasing three weeks post-surgery and remaining significantly elevated throughout the study (Fig. 3A). By week 10, the IOP of OS reached 15.2 ± 1.5 mmHg in the vehicle group, 15.2 ± 1.4 mmHg in the 2.5 µg GFAP mAb group, 15.1 ± 0.5 mmHg in the 25 µg GFAP mAb group, and 15.7 ± 0.6 mmHg in the 50 µg GFAP mAb group. These values were markedly higher than those in the untreated OD control, which maintained a stable IOP of 10.1 ± 0.3 mmHg (*p* < 0.0001).

OCT analysis revealed a progressive RNFL loss following EVO (Fig. 3B). By week 10, the RNFL thickness in the vehicle group decreased to 74.9 ± 9.0%, significantly lower than the control (99.8 ± 3.2%, *p* < 0.0001). Notably, GFAP mAb treatment attenuated RNFL loss in a dose-dependent manner, with RNFL thickness in the 2.5 µg, 25 µg, and 50 µg GFAP mAb-treated OS at 82.1 ± 7.2%, 86.5 ± 7.2%, and 85.2 ± 6.2%, respectively (*p* = 0.22, *p* = 0.013, and *p* = 0.035 vs. vehicle) (Fig. 3B and C).

### ERG-based evaluation of the effect of GFAP mAb in EVO-induced retinal dysfunction

To investigate the IOP-induced retinal function impairment, we conducted a photopic ERG. Simultaneous recordings from the OS and OD were performed, with the OD serving as a control. Quantification of b-wave and PhNR amplitudes was conducted for each group, calculating the microvolt deficit of OS relative to OD as a percentage difference (Δ = [difference / OD value] × 100%). At week 0, no significant differences were observed in b-wave or PhNR amplitude between the OS and OD across all four groups (supplementary Fig. 1).

**Fig. 4:**
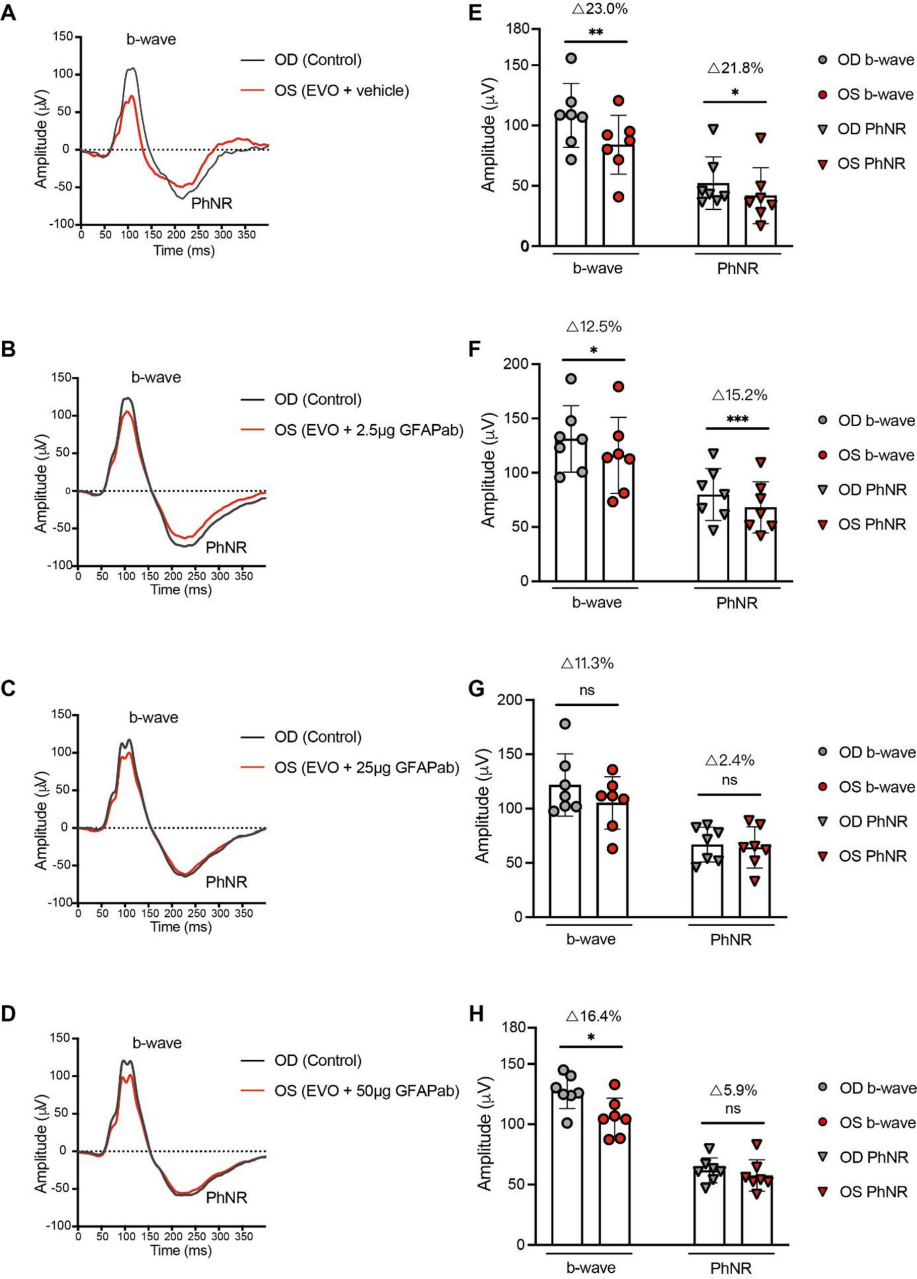
Ganzfeld ERG was performed at week 10 and the ERG pattern stimulated using a flash intensity of 1.37 log_10_ cd·s·m^− 2^ with the corresponding quantification of b-wave and PhNR amplitudes are shown. (**A**-**D**) Representative ERG recordings from OS and OD in each group in week 10 post-EVO. (**E**-**H**) The corresponding quantifications showed that the b-wave amplitudes of the OS were significantly lower than those of the OD, in the vehicle, 2.5 µg and 50 µg GFAP mAb groups; whereas this difference was not significant in the 25 µg GFAP mAb group. The PhNR amplitudes of the OS were significantly lower than those of the OD in the vehicle and 2.5 µg GFAP mAb groups, with no significant difference observed in the 25 µg and 50 GFAP mAb µg groups. Data are presented as the mean ± SD. Statistical analysis was performed using paired t-tests, with Holm-Šidák’s correction applied across groups. *p < 0.05, **p < 0.01, ***p < 0.001; ns = not significant; *n* = 7 per group

At week 10, simultaneous assessments of PhNR amplitude, an indicator of RGC activity, revealed significant impairment in the OS of the vehicle group. The PhNR amplitude in the OS was significantly reduced compared to the OD (Δ = 21.8%, *p* = 0.010, Fig. 4E). A similar decline was observed in the 2.5 µg GFAP mAb group, where the PhNR amplitude in the OS was 12.5% lower than the OD (*p* = 0.0005, Fig. 4F). These differences remained statistically significant after Holm-Šidák correction for multiple comparisons. In contrast, the 25 µg and 50 µg GFAP mAb groups showed much less impairment, with no significant difference observed between the OS and OD (Δ = 2.4% and Δ = 5.9%, *p* = 0.709 and *p* = 0.580, respectively; Fig. 4 G, H).

Similarly, a significant decline in b-wave amplitude (23.0%, *p* = 0.001, Fig. 4E) was observed in the OS compared to the OD in vehicle groups at week 10. A similar decline in b-wave amplitude was seen in the 2.5 µg and 50 µg GFAP mAb groups, where the OS amplitude was 12.5% and 16.4% lower than that of the OD, respectively (*p* = 0.011 and *p* = 0.010, Fig. 4F, H). These significant b-wave reductions were confirmed by Holm-Šidák correction for multiple comparisons. In contrast, no significant difference in b-wave amplitude between the OS and OD was observed in the 25 µg GFAP mAb group (Δ = 11.3%, *p* = 0.207, Fig. 4G).

Overall, these results suggest that treatment with GFAP mAb at higher doses (25 µg and 50 µg) helped to preserve retinal function, particularly at the 25 µg dose showing the most preservation of both PhNR and b-wave amplitudes.

### GFAP mAb promotes RGC survival in glaucomatous retina

**Fig. 5:**
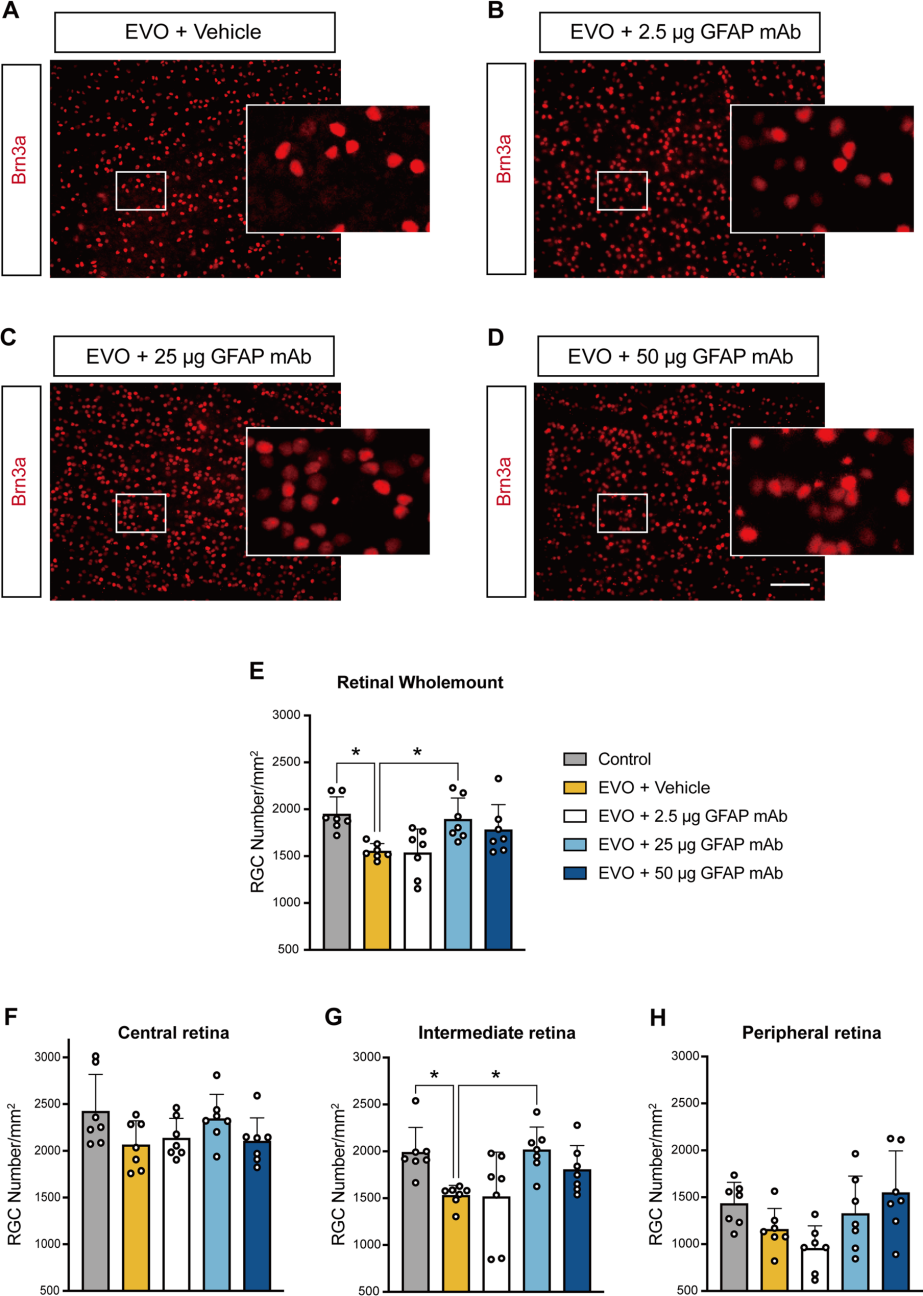
GFAP mAb promotes RGC survival in EVO-induced glaucoma. (**A**-**D**) Representative immunostaining images of Brn3a-positive RGCs in the indicated group (Scale bar = 100 μm). Images from selected regions (white squares) are shown at higher magnification. (**E**) Quantification of the mean RGC density across the entire retinal flat mount. (**F**-**H**) Quantification of RGC density in the three regions (central, intermediate, peripheral) of the retina. Data were obtained from the average of three sampling sites per region in each retina flat mount. Data are presented as the mean ± SD. *p < 0.05; one-way ANOVA followed by Tukey’s post hoc test; *n* = 7 per group

To evaluate RGC survival, we quantified RGC density in post-mortem retina flat mounts using Brn3a immunostaining, a specific marker of RGCs. The RGC density across the entire flat mount was calculated as the average of nine images per retina. The mean RGC density across the entire flat mount was 1552 ± 81 cells/mm² in the vehicle group, which was significantly lower than that of the control (1947 ± 184 cells/mm², *p* = 0.014). The 2.5 µg GFAP mAb treatment did not preserve RGC density (1534 ± 255 cells/mm²). However, treatment with 25 µg GFAP mAb significantly rescued RGC density (1892 ± 228 cells/mm², *p* = 0.044 vs. vehicle), while the 50 µg GFAP mAb group showed an improved density of 1779 ± 270 cells/mm² (*p* = 0.301 vs. vehicle) (Fig. 5E).

To investigate the region-specific protective effects of GFAP mAb, we quantified RGCs in three distinct regions (central, intermediate, and peripheral) based on their distance from the optic nerve head and calculated the mean density of three images from each region of the retina. In the central and peripheral regions, no significant differences were observed among the groups (Fig. 5F, H). However, in the intermediate region, the density of RGCs in the vehicle group was significantly reduced compared with that in the OD control group (1512 ± 109 cells/mm², *p* = 0.049). This reduction was significantly reversed by 25 µg GFAP mAb treatment (2015 ± 243.5 cells/mm², *p* = 0.033 compared to the vehicle group)(Fig. 5G). These results suggest that GFAP mAb treatment, particularly at 25 µg, effectively preserves RGC density, with the most significant protective effects observed in the intermediate region of the retina.

### GFAP mAb inhibited retinal astrocyte activation in a dose-dependent manner, accompanied by non-parallel alterations in microglial activation

**Fig. 6:**
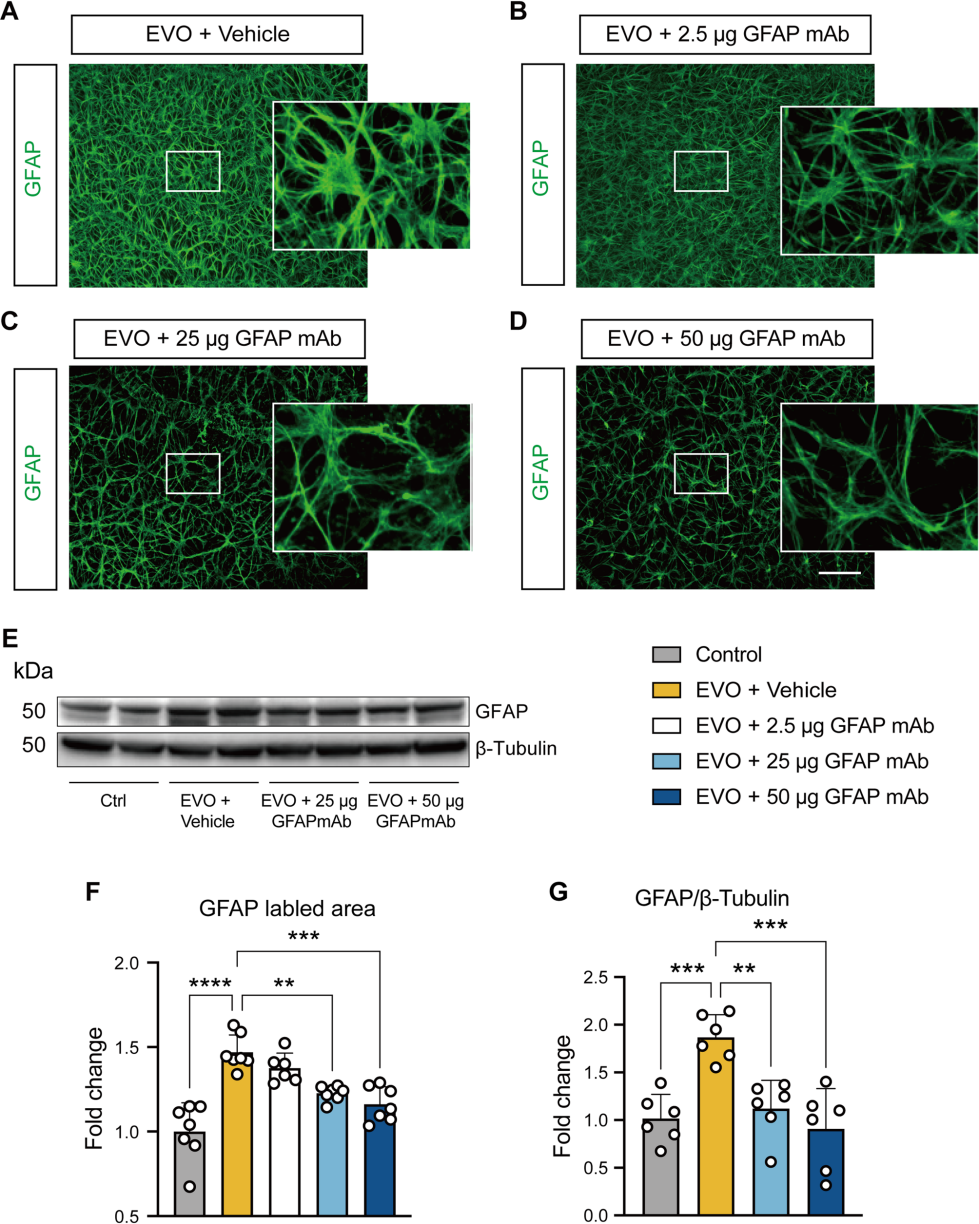
GFAP mAb treatment reduces astrocyte activation in glaucomatous retinas in a dose-dependent manner. (**A**-**D**) Representative fluorescence images of GFAP immunostaining, visualizing astrocytes in retinal flat mounts treated with vehicle, 2.5 µg, 25 µg, or 50 µg GFAP mAb (scale bar = 100 μm). Higher magnification views of selected regions (white squares) highlight morphological changes. Treatment with 25–50 µg GFAP mAb reversed the hypertrophic and ramified morphology characteristic of activated astrocytes observed in the vehicle-treated group, restoring a more organized astrocyte structure. (**E**) Representative Western blot image of GFAP, with β-Tubulin as a loading control. (**F**) Quantification of GFAP-labeled area using ImageJ. The results show that the GFAP-labeled area significantly increased in the EVO + vehicle group compared to the control, which was reduced by the treatment with 25 µg and 50 µg GFAP mAb (*n* = 7 per group). (**G**) Quantitative analysis reveals that GFAP expression increased in vehicle-treated glaucomatous retinas, but was markedly downregulated by 25–50 µg GFAP mAb. (*n* = 6 per group). Data were normalized to the control group, expressed as fold change, and are presented as mean ± SD. **p < 0.01, ***p < 0.001, ****p < 0.0001; one-way ANOVA followed by Tukey’s post hoc test

To determine the glial cell activation, the rat retinal flat mounts were immunostained for the astrocyte marker, GFAP. In the vehicle-treated group, astrocytes displayed hypertrophic and ramified morphology characteristic of a reactive phenotype (Fig. 6A), with a significant increase in the GFAP-labeled area compared to the control group (*p* < 0.0001; Fig. 6F), indicating a higher level of astrocyte activation overall, consistent with previous reports [16]. In contrast, the astrocytes in the 25 µg and 50 µg GFAP mAb-treated groups exhibited reduced branching and well-organized structure (Fig. 6C and D). Correspondingly, the GFAP-labeled area was significantly decreased with GFAP mAb treatment in a dose-dependent manner, compared to the vehicle group (25 µg: *p* = 0.002; 50 µg: *p* = 0.0001). However, treatment with 2.5 µg GFAP mAb showed no significant difference from the vehicle (Fig. 6B).

To further validate the inhibitory effect of 25–50 µg GFAP mAb treatment on astrocyte activation, we utilized Western blot to investigate GFAP expression level, as its overexpression is considered a hallmark of astrogliosis. The result revealed a significant increase in GFAP protein levels in the vehicle group compared to control (*p* = 0.0007) (Fig. 6E, G). Notably, treatment with 25–50 µg of GFAP mAb led to significant suppression of GFAP expression (*p* = 0.0025 and *p* = 0.0002 vs. vehicle, respectively), aligning with the observed reduction in the GFAP-labeled area.

**Fig. 7:**
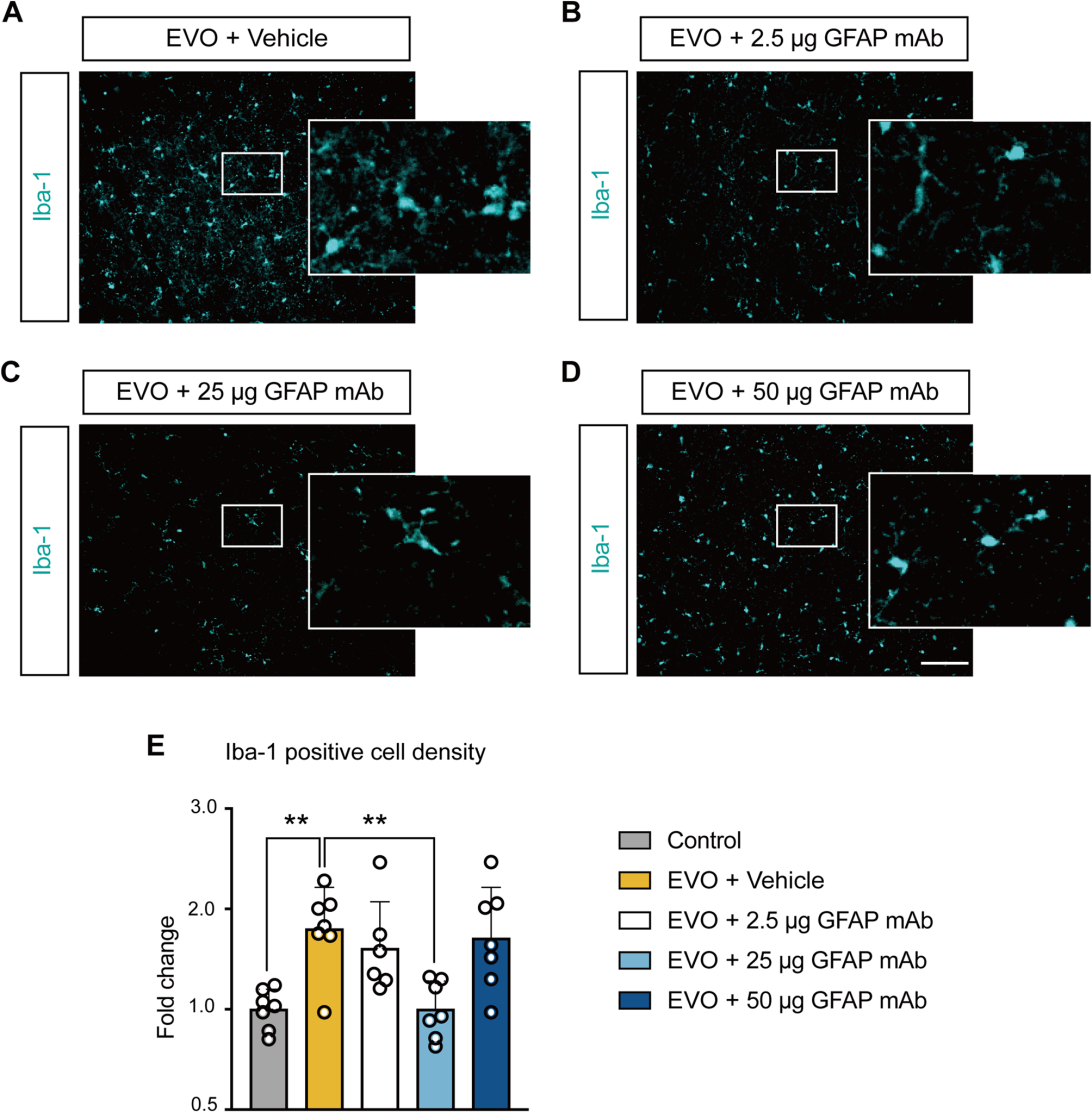
GFAP mAb modulates microglial density in glaucomatous retinas. (**A**-**D**) Representative immunofluorescence images of Iba1-positive microglia in retinal flat mounts (scale bar = 100 μm). The selected regions (white squares) are displayed at higher magnification. (**E**) Quantification of Iba1-positive cell density reveals a significant increase in microglial density in the EVO + vehicle group compared to controls. Treatment with 25 µg GFAP mAb significantly reduced microglial density, whereas 50 µg GFAP mAb failed to suppress the increase. Data were normalized to the control, expressed as fold change, and presented as mean ± SD. **p < 0.01; one-way ANOVA followed by Tukey’s post hoc test; *n* = 7 per group

Given the critical role of astrocyte-microglia interactions in the pathogenesis of glaucoma, we sought to clarify microglial alteration in response to GFAP mAb-inhibited astrogliosis. Microglia were visualized using Iba1 immunostaining, which is commonly used to identify microglia in retina (Fig. 7A-D). Although Iba1 can also label other CNS macrophage populations [28], the chronic nature of EVO model used in this study, characterized by minimal systemic immune cell infiltration [29], suggests that the Iba1-positive cells primarily represent resident microglia in this context. Microglial density was quantified and expressed as fold change relative to the control group (Fig. 7E). The vehicle group showed a significant increase in microglia density, with a fold change of 1.802 ± 0.41 compared to controls (*p* = 0.005). The treatment with 2.5 µg GFAP mAb failed to yield significant alteration. In the 25 µg GFAP mAb-treated group, microglial density significantly decreased compared to the vehicle group (fold change: 1.00 ± 0.28, *p* = 0.005). However, microglial density rebounded in the 50 µg GFAP mAb group (fold change: 1.71, *p* > 0.05), despite the further reduction of astrogliosis (as shown in Fig. 6).

In short, GFAP mAb exerts non-parallel inhibitory effects on astrocyte and microglial activation, with 25 µg GFAP mAb inhibiting both, while 50 µg GFAP mAb only inhibiting astrocyte activation.

### NF-κB and p38 MAPK pathways were inhibited following GFAP mAb treatment in glaucomatous retina

**Fig. 8:**
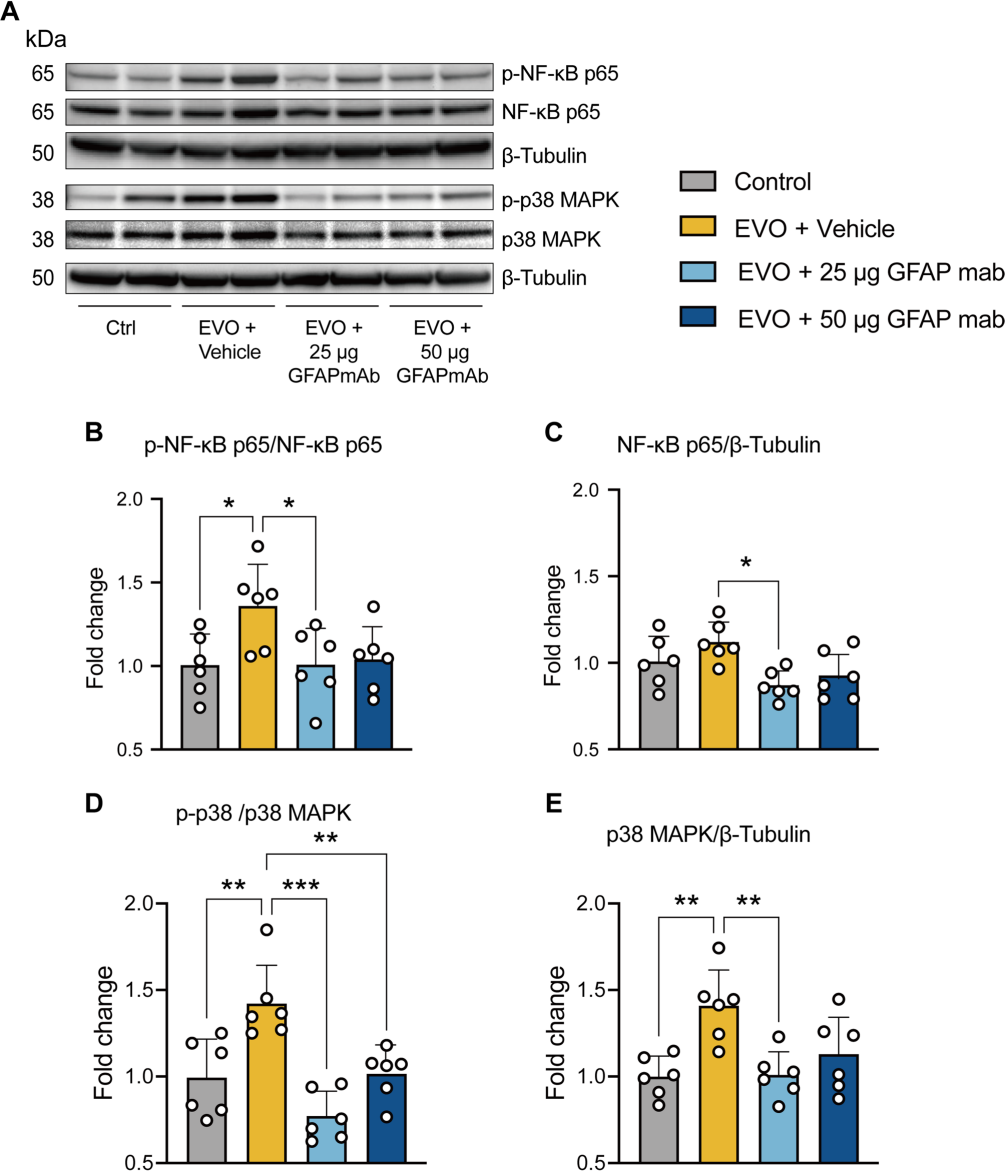
GFAP mAb inhibited the activation of p38 MAPK and NF-κB pathways in glaucomatous retinas. (**A**) Representative Western blot images illustrate the detection of the targeted proteins, with β-Tubulin as a loading control. (**B-C**) EVO-induced NF-κB p65 phosphorylation level was significantly reversed by 25 µg GFAP mAb treatment, which also downregulated NF-κB p65 expression. (**D**) Both 25 µg and 50 µg GFAP mAb significantly suppressed p38 MAPK phosphorylation induced by EVO. (**E**) Treatment with 25 µg GFAP mAb reversed p38 MAPK expression induced by EVO. Data are normalized to the control and presented as the mean ± SD. *p < 0.05, **p < 0.01, ***p < 0.001; one-way ANOVA followed by Tukey’s post hoc test; *n* = 6 per group

The activation of the p38 mitogen-activated protein kinase (MAPK) and nuclear factor-κB (NF-κB) signaling pathways play a critical role in astrocyte-mediated neurotoxicity [30, 31]. We conducted further analysis to evaluate the modulation of these pathways following GFAP mAb treatment (Fig. 8A). Immunoblot analysis showed that the vehicle-treated retinas exhibited increased activation of the NF-κB pathway, as indicated by a higher phosphorylated NF-κB p65/p65 ratio compared to controls (*p* = 0.043). The 25 µg GFAP mAb treatment significantly reduced NF-κB p65 phosphorylation and expression compared to the vehicle (*p* = 0.045 and *p* = 0.011, respectively) (Fig. 8B and C). Additionally, both p38 MAPK levels and phosphorylation levels were significantly increased in the vehicle group compared to the control (*p* = 0.003 and *p* = 0.004, respectively). Treatment with 25–50 µg GFAP mAb effectively decreased p38 MAPK phosphorylation compared to the vehicle (*p* < 0.0001 and *p* = 0.006, respectively), and p38 MAPK levels were significantly downregulated with 25 µg GFAP mAb (*p* = 0.004 vs. vehicle; Fig. 8D and E).

### Microarray-based protein analysis revealed the anti-inflammation effect of GFAP mAb

**Fig. 9:**
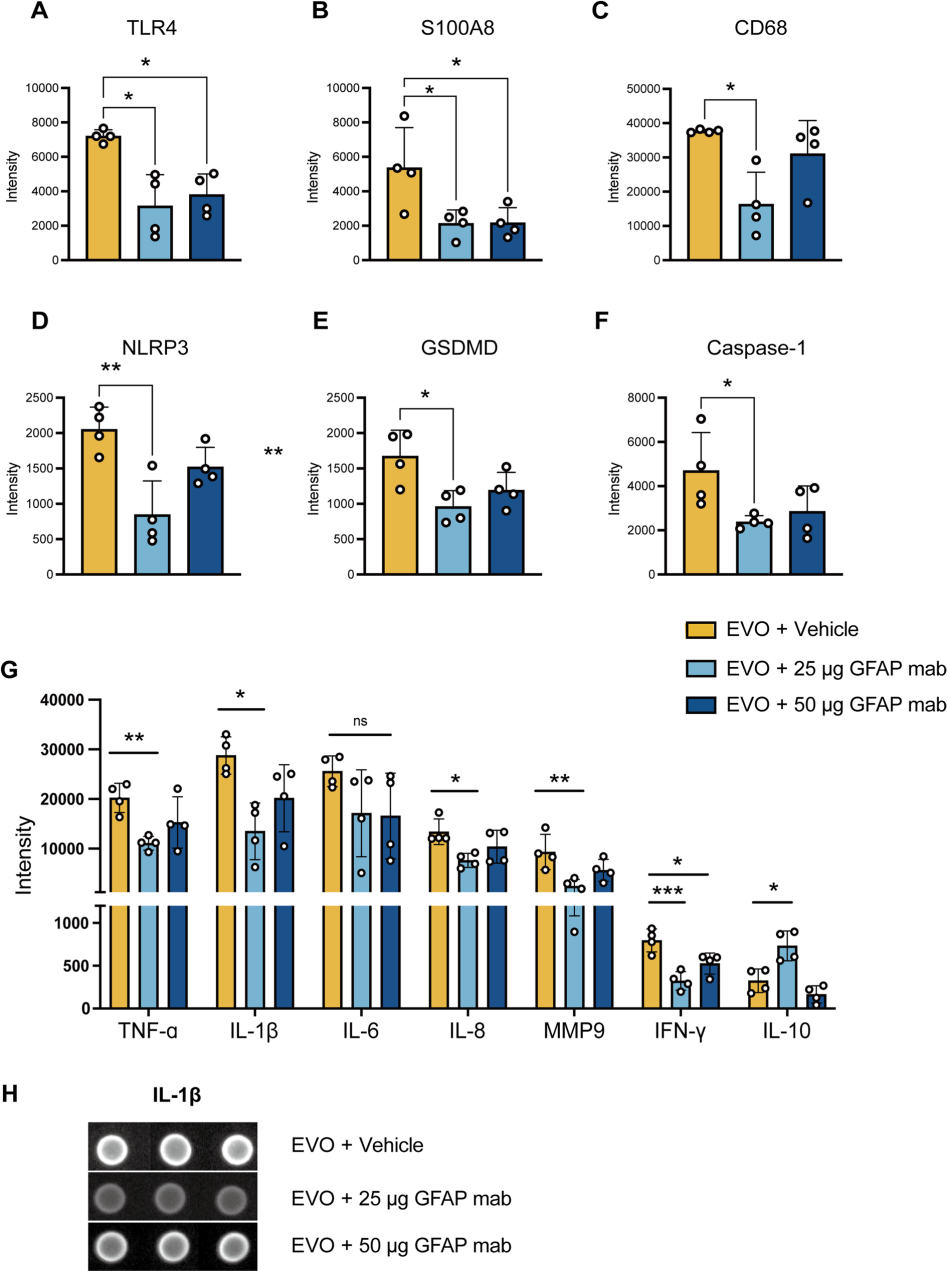
Microarray analysis reveals the effects of GFAP mAb treatment on key inflammatory mediators in glaucomatous retina. (**A**-**C**) The expression of TLR4 and S100A8 was significantly downregulated by 25–50 µg GFAP mAb treatment compared to the vehicle. The marker for microglial activation, CD68, was significantly decreased in the 25 µg GFAP mAb treatment group. (**D**-**F**) Proteins of the inflammasome pathway, NLRP3, GSDMD and Caspase-1 were significantly downregulated in the 25 µg GFAP mAb treatment group. With the 50 µg dose, these protein expressions showed a similar downward trend, but without statistical significance. (**G**) Expression profiling of inflammation-associated mediators showed that 25 µg GFAP mAb significantly decreased pro-inflammatory factors (TNF-α, IL-1β, IL-8, MMP9, and IFN-γ), and increased the anti-inflammatory cytokine IL-10 compared to the vehicle group. In contrast, the 50 µg dose significantly reduced IFN-γ only. (**H**) Representative images of spots showing IL-1β levels in the subarrays for each group. Image analysis was conducted with Imagene software, using the median intensity from each spot to calculate the mean of the triplicate spots for each marker. For CD68 and TLR4, due to significant heterogeneity in SDs, statistical analysis was performed using Welch’s ANOVA followed by the Tamhane T2 post hoc test. All other data were analyzed using one-way ANOVA with Tukey’s post hoc test. Data are presented as the mean ± SD; *n* = 4 per group; *p < 0.05, **p < 0.01, ***p < 0.001; ns = not significant. TLR4 – Toll-Like Receptor 4. S100A8 – S100 Calcium Binding Protein A8. NLRP3 – NLR Family Pyrin Domain Containing 3. GSDMD – Gasdermin D

We evaluated the impact of GFAP mAb on neuroinflammation, given its potent effects on gliosis modulation and the inhibition of the NF-κB and p38 MAPK pathways as described above. High-throughput microarray analysis was applied to profile neuroinflammation-related protein expression (Fig. 9). The findings revealed that neuroinflammatory mediators, including the Toll-like receptor 4 (TLR4) and the S100 calcium-binding protein A8 (S100A8), were downregulated by 25 µg (*p* = 0.039 and *p* = 0.026 vs. vehicle) or 50 µg GFAP mAb treatment (*p* = 0.010 and *p* = 0.027 vs. vehicle) (Fig. 9A and B). This reduction paralleled the observed inhibition of astrogliosis. The analysis of CD68, a marker for microglial activation, demonstrated a significant decrease in expression with 25 µg GFAP mAb treatment (*p* = 0.034 vs. vehicle), though not with the 50 µg dose (Fig. 9C), which aligns with the observed modulation in microglial density.

Further analysis of the inflammasome pathway showed that NLR family pyrin domain-containing 3 (NLRP3), gasdermin D (GSDMD) and Caspase-1, were significantly downregulated in the 25 µg GFAP mAb group compared to the vehicle (*p* = 0.002, *p* = 0.013 and *p* = 0.044 respectively, Fig. 9D-F). Although the 50 µg GFAP mAb group also exhibited a downward trend in the expression of these proteins, the changes did not reach statistical significance compared to the vehicle.

Moreover, the downstream factors, including both pro- and anti-inflammatory cytokines such as tumor necrosis factor-alpha (TNF-α), interferon-gamma (IFN-γ), interleukin-1 beta (IL-1β), interleukin-6 (IL-6), interleukin-8 (IL-8), interleukin-10 (IL-10), as well as matrix metalloproteinase-9 (MMP9), which is involved in extracellular matrix remodeling and inflammation, were also investigated. Compared to the vehicle group, treatment with 25 µg GFAP mAb significantly reduced levels of pro-inflammatory factors: TNF-α (*p* = 0.009), IL-1β (*p* = 0.033), IL-8 (*p* = 0.020), MMP9 (*p* = 0.007), and IFN-γ (*p* = 0.0008), while significantly increasing the anti-inflammatory cytokine IL-10 (*p* = 0.019) (Fig. 9G). In contrast, the 50 µg dose only significantly reduced IFN-γ (*p* = 0.02 vs. vehicle), with no significant effects on other factors.

These results indicates that treatment with 25 µg GFAP mAb inhibited microglial activation, and yielded broad suppression of neuroinflammation-related mediators, significantly reducing key pro-inflammatory mediators and upregulating the anti-inflammatory cytokine IL-10; while the 50 µg GFAP mAb dose only led to significant reductions in TLR4, S100A8, Caspase-1 and IFN-γ.

### GFAP mAb prevented R28 cell death in an in vitro degeneration model

**Fig. 10:**
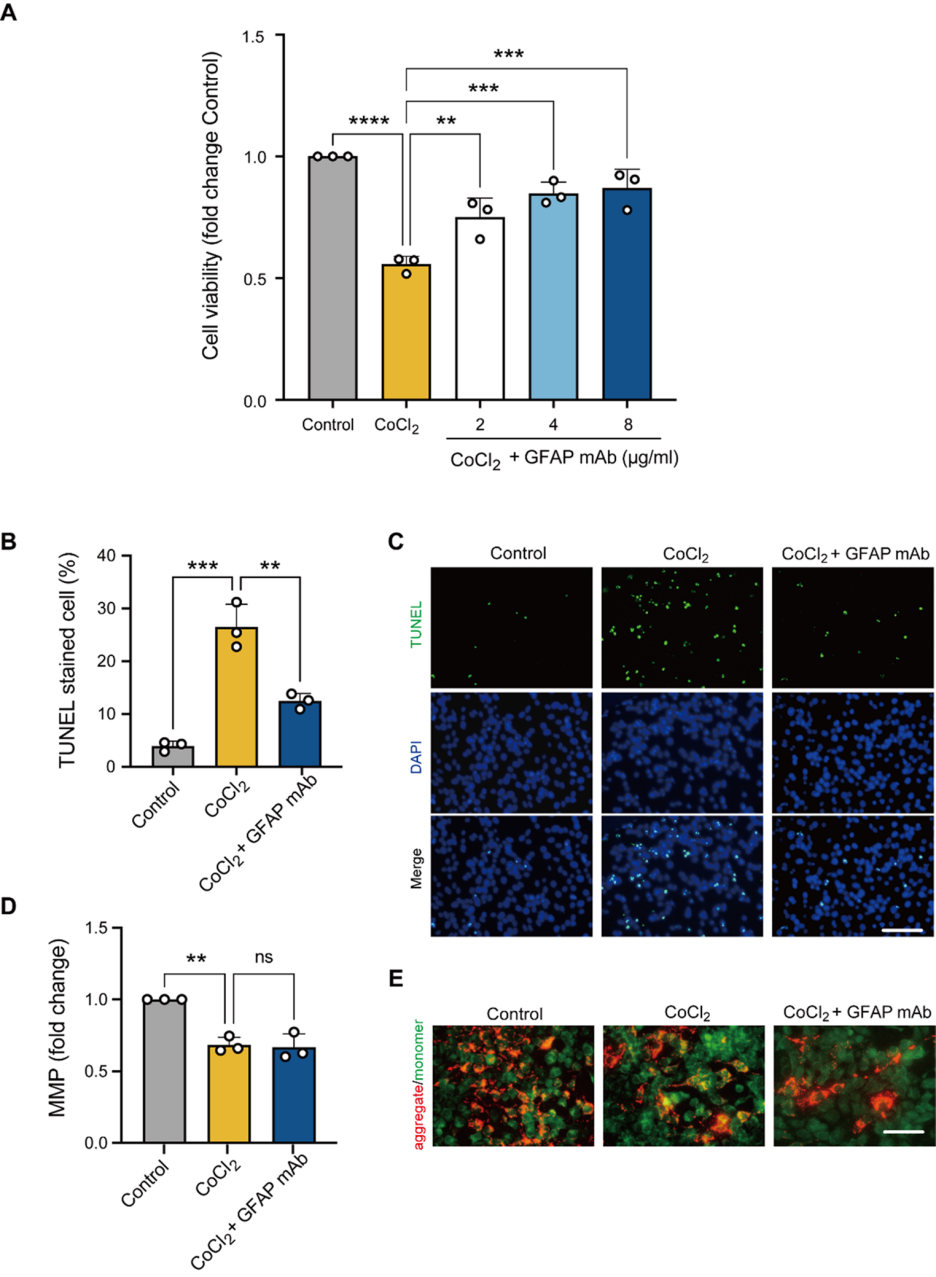
GFAP mAb treatment protects R28 cells from CoCl_2_-induced cell death in an in vitro degeneration model. (**A**) Cell viability was assessed using an MTS assay. After exposure to 50 µM CoCl_2_, R28 cells were treated with GFAP mAb at the indicated concentrations (2, 4, 8 µg/ml) for 24 h, resulting in a significant concentration-dependent improvement in the cell viability. (**B**- **C**) Quantitative analysis of TUNEL-positive cells and representative images of TUNEL and DAPI staining show that the GFAP mAb (8 µg/ml) treatment significantly reduced the number of TUNEL-positive cells induced by CoCl_2_ stress. Scale bar = 100 μm. (**D)** Quantitative analysis of MMP, represented by the red-to-green fluorescence ratio, revealed that GFAP mAb treatment failed to alleviate CoCl_2_-induced MMP impairment. **(E**) Representative images showing JC-10 monomer (green), aggregate (red), and their merged overlap (orange) following CoCl₂ exposure and GFAP mAb treatment. Scale bar = 100 μm. Data are expressed as the mean ± SD from three independent experiments. **p < 0.01, ***p < 0.001 and ****p < 0.0001, ns = not significant; one-way ANOVA with Tukey’s post-hoc test

To confirm the protective effects of GFAP mAb, we tested its efficacy in vitro using an R28 retinal cell degeneration model, with cobalt chloride (CoCl_2_) stimulation. To determine the optimal concentration of GFAP mAb, R28 cells were exposed to 50 µM CoCl_2_ for 24 h, followed by treatment with GFAP mAb at concentrations ranging from 2 to 8 µg/ml for another 24 h. The cell viability was then assessed using the MTS assay. As shown in Fig. 10A, GFAP mAb treatment improved the viability of R28 cells in a concentration-dependent manner. The 8 µg/ml dose yielded the highest R28 viability (*p* = 0.0002, vs. CoCl_2_), and this concentration was subsequently used in further experiments.

The effect of GFAP mAb on cell death was then investigated using the TUNEL assay. R28 cells were subjected to 50 µM CoCl_2_ for 24 h, followed by treatment with or without 8 µg/ml GFAP mAb for another 24 h. The results showed that CoCl_2_ exposure significantly increased the percentage of TUNEL-positive cells compared to the control (3.92 ± 0.91% vs. 26.45 ± 4.32%, *p* = 0.0001), whereas GFAP mAb treatment substantially reduced this proportion to 12.45 ± 1.44% (*p* = 0.002 vs. CoCl_2_) (Fig. 10B and C). Next, we examined its potential effect on mitochondrial function, using a JC-10 assay to evaluate the MMP level. MMP is a global indicator of mitochondrial function in response to oxidative stress. The results showed a significant decline in MMP following CoCl_2_ exposure (*p* = 0.002 vs. control), but GFAP mAb treatment did not restore MMP levels in hypoxic R28 cells (Fig. 10D and E). These findings suggest that GFAP mAb reduces cell death independent of direct mitochondrial preservation.

### Inhibition of pyroptosis involved in the cytoprotective mechanism of GFAP mAb

**Fig. 11:**
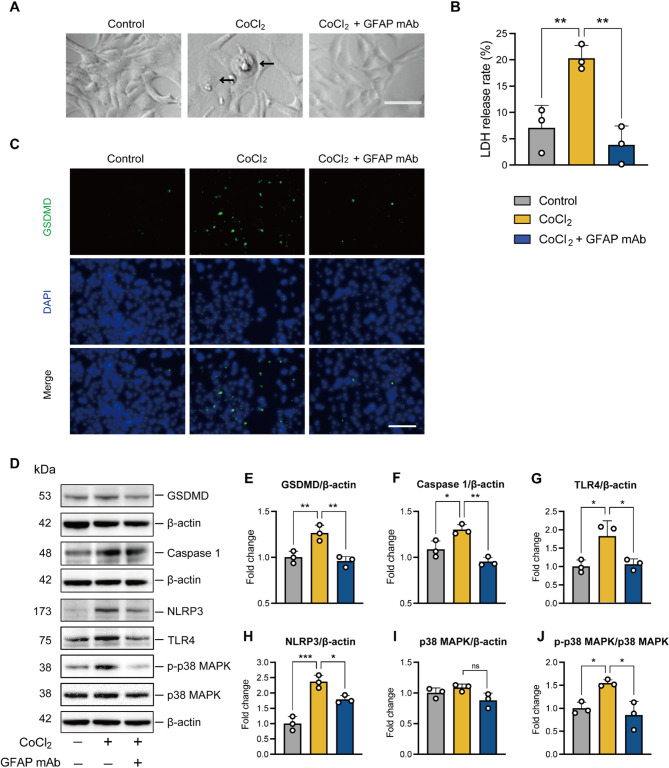
GFAP mAb confers cytoprotection to R28 cells against hypoxia-induced stress in vitro by inhibiting pyroptosis. (**A**) Representative microscopy bright field images of R28 cells under the indicated condition. The GFAP mAb treatment reversed the swelling and bubble-like protrusions morphology changes (black arrow) induced by CoCl_2_. Scale bar = 50 μm. (**B**) The LDH assay demonstrated that GFAP mAb treatment significantly reduced CoCl_2_-induced LDH release in R28 cell culture medium. (**C**) Immunostaining for GSDMD in R28 cells showed that the CoCl_2_-induced GSDMD-positive cells were decreased following GFAP mAb treatment. Scale bar = 100 μm. (**D**) Representative Western blot images of the indicated proteins, with β-actin serving as the loading control. (**E**-**J**) Western blot quantification shows that CoCl_2_ exposure upregulated GSDMD, NLRP3, and Caspase-1, which were downregulated following GFAP mAb treatment. GFAP mAb also downregulated TLR4 expression and inhibited the phosphorylation of p38 MAPK, as shown by the p-p38 MAPK to p38 MAPK ratio. The fold change in protein expression levels relative to control is presented. Data are expressed as the mean ± SD from three independent experiments. *p < 0.05, **p < 0.01, ***p < 0.001. ns = not significant; one-way ANOVA with Tukey’s post-hoc test

To elucidate the cytoprotective mechanism of GFAP mAb, we examined its effect on pyroptosis, a newly identified form of programmed cell death characterized by the activation of inflammation. It involves the formation of pores in the plasma membrane, which function as nonselective channels, allowing the influx of water and different ions. This continuous influx of water leads to cell swelling, ultimately resulting in the rupture of the plasma membrane [32, 33]. In this study, we observed that R28 cells exposed to CoCl_2_ exhibited characteristic morphological features of pyroptosis including swelling morphology and bubble-like plasma membrane protrusions. In contrast, GFAP mAb treatment reversed these CoCl2-induced morphological changes (Fig. 11A). LDH release has been widely used to monitor pyroptosis, indicating loss of membrane integrity and cell lysis [34]. As shown in Fig. 11B, LDH release was significantly increased in R28 cells after CoCl_2_ exposure compared to the control group (*p* = 0.009). In contrast, R28 cells treated with GFAP mAb exhibited a substantial reduction in LDH release compared to the CoCl_2_ group (*p* = 0.003). To further confirm the involvement of pyroptosis, we examined the expression of pyroptosis-related proteins (Fig. 11C- J). Our results demonstrated that GSDMD, a key executioner of pyroptosis, was significantly upregulated following CoCl_2_ exposure (*p* = 0.007 vs. control). However, this expression was reversed by GFAP mAb (*p* = 0.004 vs. CoCl_2_) (Fig. 11C, E). Next, we investigated the canonical pyroptosis pathway-related proteins, NLRP3 and Caspase-1. The results demonstrated that CoCl_2_ exposure significantly upregulated both NLRP3 and Caspase-1 (*p* = 0.0003 and *p* = 0.020 vs. control); however, this upregulation was effectively suppressed by GFAP mAb treatment (*p* = 0.02 and *p* = 0.002 vs. CoCl_2_) (Fig. 11F and H). Furthermore, Western blot analysis revealed a decrease in TLR4 expression (*p* = 0.048 vs. CoCl_2_) and reduced phosphorylation of p38 MAPK (*p* = 0.011 vs. CoCl_2_) in R28 cells treated with GFAP mAb under CoCl₂-induced stress (Fig. 11G, I, and J). Collectively, these findings suggest that the cytoprotective effects of GFAP mAb are associated with the inhibition of NLRP3/Caspase-1-mediated pyroptosis, potentially through the modulation of TLR4 and p38 MAPK signaling pathways.

### Bioinformatic analysis of the protein expression profiles in glaucoma rat retina

**Fig. 12:**
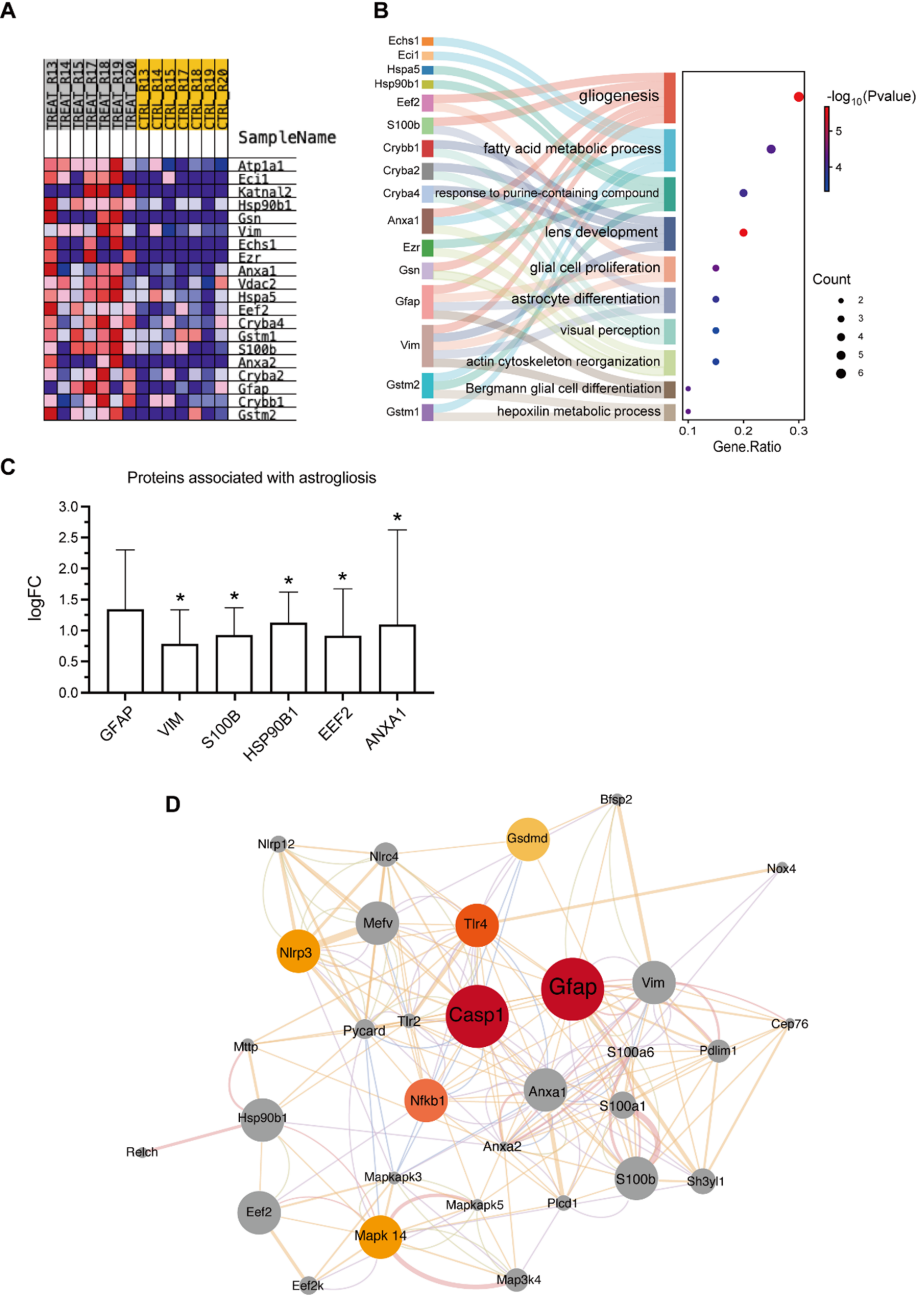
Bioinformatic analysis of EVO-induced glaucoma retina expression profiles and putative mechanism network of GFAP mAb. (**A**) Heatmap of the top 20 feature genes enriched in the EVO-treated group, identified by GSEA. (**B**) Sankey diagram combined with a bubble plot showing statistically enriched GO terms for the top 20 feature genes in biological processes. Pathways related to gliogenesis, glial cell proliferation, and astrocyte differentiation were significantly enriched in glaucomatous retinas. (**C**) Overexpression of gliosis-associated genes (GFAP, VIM, S100B, HSP90B1, EEF2, and ANXA1) in glaucomatous retinas compared to controls (*p < 0.05). A LogFC > 0 indicates protein upregulation. (**D**) Protein-protein interaction network representing the putative mechanism of GFAP mAb action. GENEMANIA output was analyzed using Cytoscape, with node size (score), node color (degree; yellow to red), and edge thickness (normalized max weight) representing interaction strength

We re-analyzed the retinal protein expression profile (PXD005258) from glaucomatous rat retinas, available in the PRIDE database. GSEA was performed on the EVO-treated and control groups to interpret the expression data in a threshold-free manner. As shown in Fig. 12A, the heatmap displays the top 20 feature genes enriched in EVO-induced glaucomatous retinas, including GFAP. GO enrichment analysis (Fig. 12B) of these genes highlighted key biological processes such as gliogenesis, glial cell proliferation, and astrocyte differentiation. In addition to GFAP, other proteins such as VIM, S100B, HSP90B1, EEF2, and ANXA1 were identified within the top 20 feature genes. Re-analysis of the PXD005258 dataset revealed that GFAP was upregulated in glaucomatous retinas compared to controls, although this difference was not statistically significant. Notably, VIM, S100B, HSP90B1, EEF2, and ANXA1 were significantly overexpressed (Fig. 12C). VIM and S100B are strongly associated with astrogliosis, as they are frequently upregulated during the reactive response of astrocytes to injury [35, 36]. HSP90B1, EEF2, and ANXA1 are involved in cellular stress responses and have been reported to be linked to gliosis-associated neuroinflammation [37–39].To predict the network-based functional implications of proteins modulated by GFAP mAb treatment, we performed a GENEMANIA bioinformatic analysis on genes corresponding to the markers identified in our Western blot studies: GFAP, CASP1, NF-κB1, MAPK14, TLR4, NLRP3, and GSDMD. Figure 12D illustrates the proposed mechanism network regulated by GFAP mAb, with GFAP and CASP1 as central nodes. Proteins regulated by GFAP mAb treatment (depicted in a gradient from yellow to red) were found to interact closely with gliosis-associated proteins. Collectively, this bioinformatic analysis, combined with our experimental findings, supports the conclusion that GFAP mAb effectively mitigates glial cell activation in glaucomatous retinas.

## Discussion

Substantial efforts have been made to develop neuroprotective therapies in glaucoma via modulating glia activation, as it has emerged as a key player in initiating and accelerating disease progression. A previous study on the autoantibody profile of glaucoma patients found a distinct loss of autoantibody targeting GFAP, which is a main type III intermediate filament protein of astrocytes [22] and considered a hallmark of astrocytes reactivity. It is still an open question if the deficiency of GFAP autoantibody is involved in dysregulated astrocyte activation during glaucoma progression. Using a well-established chronic glaucomatous rat model, we showed a pathological mechanism whereby OHT promotes astrogliosis with overexpressed GFAP protein, and blocking of which with a GFAP mAb alleviated the activation of astrocytes. Here, we postulated a new perspective in the sense of supplementing GFAP antibody to confer neuroprotection in glaucoma. Our results showed that GFAP mAb treatment mitigated glaucomatous neurodegeneration both structurally and functionally in vivo, by modulating astrogliosis and astrocyte-microglia activation to inhibit neuroinflammation in glaucoma, ultimately promoting RGC survival. In addition, we performed in vitro experiments to investigate the effects of GFAP mAb at the cellular level. GFAP mAb significantly protected retinal progenitor R28 cells. Our mechanistic analysis revealed that this was achieved particularly via inhibiting molecular responses that regulate an inflammatory form of cell death, known as pyroptosis.

In most mammals, retinal astrocytes are mainly located in the ganglion cell layer (GCL) and the nerve fiber layer (NFL) [40], where they may play an initial defensive role. In glaucomatous retinas, astrocytes formed a honeycomb plexus arranged parallel to the retinal surface [16]. These cells are highly sensitive to IOP elevation and respond rapidly with structural and molecular changes [41], intended to isolate neighboring neurons from the mechanical insult program to minimize the initial damage [42]. However, sustained astrocyte reactivation progresses to astrogliosis, which exerts deleterious effects through the release of neurotoxic molecules, such as proinflammatory cytokines [43], nitric oxide [44], and excess glutamate [45, 46], ultimately triggering RGC degeneration. These astrocyte-derived factors also stimulate microglia, further propagating neuroinflammation [47]. Reactive astrogliosis is marked by increased astrocyte proliferation, enlarged cell morphology with extended processes, and upregulation of GFAP [8]. GFAP, a key component in the assembly and extension of IFs within astrocytic processes [7, 9], is essential for the structural integrity and maintenance of activated astrocytes [48]. Previous studies have evidenced the pivotal role of GFAP in astrogliosis by promoting cytokines release [49] or directly controlling astrocyte proliferation [7]. These effects can be mitigated through pharmacological or genetic modulations of GFAP [7, 50]. In this study, astrogliosis was induced in glaucomatous rat retina, as evidenced by increased GFAP immunostaining coverage, characteristic morphological changes in astrocytes, and elevated GFAP protein levels (Fig. 6). Notably, GFAP mAb effectively inhibited astrogliosis in a dose-dependent manner, reducing GFAP protein level and the GFAP-labeled area, while reversing astrocytic morphology in glaucomatous retinas. These results are consistent with findings from genetic studies in which GFAP ablation significantly reduced astrocytic process and glial scarring [50]. Although the precise mode of action remains to be fully clarified, our findings suggest that GFAP mAb may inhibit GFAP filament assembly or promote its destabilization. This effect resembles the action of Withaferin-A, a small molecule that modifies GFAP at Cys-294, disrupting intermediate filament formation and reducing GFAP expression [51]. While the antibody likely acts through a different mechanism than WF-A, the observed phenotypic similarities suggest that GFAP mAb may modulate GFAP function by blocking polymerization or facilitating destabilization. Further studies, including epitope mapping and mechanistic assays in primary astrocytes, are needed to define the antibody’s interaction with GFAP more precisely.

In this study, the GFAP mAb treatment delayed the progression of glaucoma neurodegeneration structurally and functionally. We performed in vivo PERG to assess retinal function, quantifying PhNR amplitude as an indicator of RGC bioelectrical activity. To address the interference of general anesthesia, which inevitably alters both retinal [52] and cortical responses [53, 54], we measured and recorded the photopic ERG of both eyes simultaneously (under identical anesthesia) in each rat, with the OD serving as the control. By the end of the experiment, the significant PhNR amplitude decline in OS compared to OD was prevented in rats treated with either 25–50 µg GFAP mAb, suggesting a preservation of RGC function. On longitudinal evaluation, OCT recorded a progressive RNFL thinning as early as three weeks after glaucoma induction, when stable ocular hypertension was achieved. Consistent with the in vitro model findings, we observed a dose-dependent protection of RNFL in vivo by GFAP mAb at week 7. It is noteworthy that the long-term (week 10) protective effect of 50 µg GFAP mAb was slightly diminished compared to 25 µg GFAP mAb (Fig. 3). Postmortem analysis also revealed that the 25 µg GFAP mAb treated group exhibited more significant RGC survival than 50 µg GFAP mAb. This suggests that astrocyte-regulated signaling is involved in the early stages of glaucomatous neurodegeneration, whereas later stages may involve additional injury mechanisms, including microglial activation.

Interestingly, while GFAP mAb dose-dependently suppressed astrogliosis, its effects on microglial activation were not parallel. Specifically, retinas treated with 25 µg of GFAP mAb not only showed suppressed astrogliosis but also a significant reversal of microglial activation, as evidenced by decreased microglial density and CD68 protein expression levels. Generally, astrocytes and microglia could be activated in a bidirectional manner through the secretion of multiple cytokines and inflammatory mediators [55]. In this study, the downregulation of GFAP protein by GFAP mAb treatment inhibited the NF-κB pathway, which is known to promote the expression of proinflammatory cytokines and chemokines [56]. Consistent with this, our protein microarray analysis indicated that 25 µg GFAP mAb treatment resulted in a broad suppression of various upstream and downstream inflammatory mediators. This suggests that disrupting the astrocyte-microglia activation axis through targeting GFAP may be an effective strategy for controlling neuroinflammation. However, compared to the 25 µg GFAP mAb-treated retinas, the 50 µg dose unexpectedly resulted in higher microglial density and CD68 expression, with no significant difference from the vehicle group, despite more pronounced inhibition of astrogliosis. Similarly, suppression of pro-inflammatory factors and NLRP3 pathway activation was less effective at the higher dose. One possible explanation is that the excessive inhibition of astrocyte activity at the 50 µg dose may impair their essential homeostatic and immunoregulatory roles, leading to compensatory microglial activation. Astrocytes play essential supportive and regulatory functions [57], including maintaining microglial homeostasis through direct contact and secreted factors such as IL-10 and TGF-β [58, 59]. Over-suppression of astrocytes may therefore disrupt this regulatory balance. Previous studies have shown that impaired astrocyte function in GFAP/Vimentin knockout models lead to increased microglial numbers [60]. Similarly, inhibition of astrocyte proliferation via soluble adenylyl cyclase blockade also elevates microglial density [61]. In GFAP knockout models, enhanced microglial proliferation and upregulation of pro-inflammatory genes have been reported, further supporting that impaired astrocyte function may promote compensatory microglial reactivity [62]. In this study, we noted a remarkably lower expression level of the anti-inflammatory cytokine IL-10 in the retina treated with 50 µg of GFAP mAb than those treated 25 µg dose, with no significant difference observed between the 50 µg group and the vehicle group (Fig. 9). Given that astrocyte-derived IL-10 is known to play a critical role in restraining microglial activation [63], this reduction may further explain the diminished neuroprotective effect at the higher dose. Together, these results suggest that excessive astrocyte suppression by high-dose GFAP mAb may undermine microglial homeostasis, diminishing the neuroprotective effect. Future studies, particularly using astrocyte-microglia co-culture systems, are warranted to validate whether high doses of GFAP mAb excessively suppress astrocytic functions and thereby induce microglial dysregulation.

GFAP mAb treatment significantly enhanced RGC survival, primarily by suppressing neuroinflammatory pathways, including p38 MAPK and NF-κB. Activation of p38 MAPK has been identified as a key regulator of neuroinflammation in central nervous system (CNS) diseases such as Alzheimer’s disease [64] and ischemic stroke [65]. In the retina, astrocyte activation has been proven to depend on p38 activation, as demonstrated in a chronic glaucoma rat model [30]. Moreover, studies have shown that specifically targeting glial IF including GFAP with withaferin A inhibits p38 pathway activation in retinal astrocytes [49]. Based on these findings, we propose that GFAP mAb targets GFAP to disrupt intermediate filament assembly, which subsequently inhibits p38 MAPK activation. Furthermore, previous studies have demonstrated that p38 MAPK regulates the transcriptional activity of NF-κB by phosphorylating p65 at S276 and acetylating p65 at K310 during inflammatory responses [66, 67], suggesting that NF-κB may function as a downstream signaling molecule.

Protein microarray analysis further revealed that 25 µg GFAP mAb had a stronger anti-inflammatory effect than the 50 µg dose, leading to the highest RGC survival rates. This dose significantly downregulated S100A8, TLR4, NLRP3, GSDMD, Caspase-1, and key inflammatory cytokines, including TNF-α, IL-1β, IL-8, MMP9, and IFN-γ. Activated astrocytes express damage-associated molecular patterns (DAMPs) like S100A8 in response to injury [68], which interact with TLR4 to activate the NLRP3 inflammasome in astrocytes and microglia [69–71]. The key role of NLRP3 inflammasome activation in glaucoma has been highlighted, leading to increased pro-inflammatory cytokine release and creating a chronic inflammatory environment in the retina, which can exacerbate RGC loss and contribute to neurodegeneration [72, 73]. Inflammatory cytokines, such as TNF-α, IL-1β and IL-6, have been identified to clearly contribute to RGC death in glaucoma [74, 75]. Prior studies also suggest that inhibiting glial-derived pro-inflammatory factors enhances RGC survival [76, 77]. Consistent with these findings, our results suggest that GFAP mAb-enhanced RGCs survival was associated with a general decrease in inflammatory cytokines. In addition, NLRP3 could be activated to oligomerization with apoptosis-associated speck-like protein containing a CARD (ASC) and pro-caspase 1, subsequently resulting in cleavage of GSDMD and pro-IL-1β, initiating pyroptosis and contributing to axon degeneration and RGC death [78, 79]. In summary, our findings indicate that GFAP mAb treatment inhibits p38 MAPK/NF-κB, reduces S100A8 release, and blocks TLR4-mediated NLRP3 inflammasome activation, leading to a reduction in pro-inflammatory cytokines and ultimately promoting RGC survival.

The in vitro analysis provided us with a more in-depth perspective to reveal the direct effects of GFAP mAb at a cellular level. The retinal precursor R28cell line expresses both glial and ganglion cell marker patterns and acts as a pluripotential precursor [80], serving as a unified model to gain supplementary mechanistic insights into GFAP mAb activity. GFAP mAb enhanced R28 cell viability under hypoxic stress in a dose-dependent manner and significantly reduced cell death by suppressing pyroptosis, as evidenced by decreased TUNEL staining, reduced LDH release, downregulation of pyroptosis-related proteins. Pyroptosis has been identified as a significant mechanism of RGC death. It is a specific inflammatory and lytic form of programmed cell death, characterized by cell swelling, membrane rupture, and massive leakage of cytosolic contents [81]. This process is primarily mediated through NLRP3/Caspase-1 (canonical pathway) and the executioner protein GSDMD [82]. Previous research has shown that activation of the NLRP3/Caspase-1 inflammasome triggers pyroptosis and contributes to high IOP-induced RGC loss [83]. Genetic deletion of GSDMD, the effector of pyroptosis, markedly ameliorated RGC death and retinal tissue damage in acute glaucoma [78]. Similar evidence suggests that hypobaric hypoxia exposure initiates pyroptosis in the rat retina, which can be reversed by inhibiting NLRP3 inflammasome activation and pro-inflammatory cytokine secretion [84]. In this study, we found that R28 cells under CoCl_2_ exposure also exhibited an upregulation of the NLRP3/Caspase-1/GSDMD pyroptosis pathway, which was substantially reversed by GFAP mAb treatment. In addition, TLR4 knockdown inhibited p38 MAPK activation, thereby reducing cell pyroptosis and inflammatory response by suppressing the secretion of IL-1β and TNFα [85]. Our results demonstrated that the phosphorylation of p38 MAPK and expression of TLR4 were blocked by the administration of GFAP mAb, suggesting that it possibly inhibited pyroptosis via modulation of the TLR4/p38 MAPK/NLRP3 pathway.

We used a bioinformatic approach to explore the putative mechanism of GFAP mAb regulation in glaucoma signature proteins. The re-analysis of a public proteomics dataset revealed that gliogenesis and astrocyte differentiation are among the most prominent pathological features of glaucoma, as shown by GO enrichment analysis of the top 20 characteristic genes in the EVO-induced glaucoma group. More importantly, the proteins regulated by GFAP mAb formed a closely connected protein interaction network with the characteristic proteins of glaucoma, in which GFAP and Caspase-1 were revealed as the central nodes (Fig. 9). This indicates that GFAP mAb may modulate the pathological protein network of glaucoma, particularly through the key regulators GFAP and Caspase-1.

While our findings highlight the neuroprotective potential of GFAP mAb in modulating astrocyte reactivity and neuroinflammation in glaucoma model, clinical translation will require addressing several challenges. These include optimizing dosing strategies to avoid disrupting astrocyte function at higher doses and ensuring efficient, localized delivery to minimize systemic exposure. Furthermore, as the GFAP mAb used in this study is of non-human origin, future work should focus on sequencing the antibody to identify binding epitopes and key functional domains, enabling the development of humanized or fully humanized versions to reduce immunogenicity risks. A comprehensive evaluation of its long-term safety and efficacy will also be necessary to establish the therapeutic profile. These efforts will be crucial for advancing GFAP mAb as a potential treatment for glaucoma and other retinal neurodegenerative diseases.

There are limitations in the current study. Only female SD rats were used in this study, which may limit the broader interpretation of sex-specific differences in disease pathogenesis and treatment responses. This choice was based on both practical and biological considerations. Female rats exhibit lower aggression generally, thereby reducing stress-induced variability that could influence the IOP [86] and inflammatory responses [87]. However, sex-related differences in IOP dynamics and disease progression may arise due to hormonal influences [88]. Nonetheless, future studies including both sexes are essential to assess potential sex-specific responses. In addition, this study focused on the effect of GFAP mAb on the overall activation of astrocytes, whereas the responses of individual astrocyte subtypes to GFAP mAb were not separately assessed. Different phenotypes of reactive astrocytes have previously been characterized, including A1-like neurotoxic and A2-like neuroprotective subtypes [89]. However, it has been suggested that reactive astrocytes diversify beyond these two simplified states [90]. Future studies analyzing specific markers of astrocytes phenotypes, potentially combined with transcriptomic or proteomic approaches, will be required to determine whether GFAP mAb has a specific inhibitory effect on neurotoxic astrocytes. Additionally, the R28 cell line, being a retinal precursor cell line with mixed neuronal and glial properties, has inherent limitations in fully reflecting the effects of GFAP mAb on glial cells, particularly astrocytes. While R28 cells express certain glial markers [91], their mixed nature may not accurately replicate the specific glial responses seen in astrocytes during injury or neuroinflammation. Therefore, the R28 cell model should be considered a supplementary tool for investigating the effects of GFAP mAb on retinal cell viability, pyroptosis, and inflammasome pathways, rather than a definitive model for glial activation. Future studies should consider using primary astrocytes or well-characterized astrocyte cell lines to better understand the specific mechanisms underlying GFAP mAb’s effects on glial activation. Secondly, the protective effects of GFAP mAb were demonstrated against neuroinflammation, hypoxic stress, and pyroptosis. However, the pathological process of glaucoma also involves other types of injury such as oxidative stress and excitotoxicity [92, 93]. Further studies using different stress models are required to fully determine the role of GFAP mAb under other neuropathological stresses, as well as the effect of GFAP mAb on different forms of cell death, such as apoptosis, necrosis, and autophagy.

## Conclusion

Collectively, this study demonstrates that targeting GFAP with a monoclonal antibody effectively suppresses astrogliosis and attenuates neuroinflammation in vivo, resulting in protection of RGCs in a glaucoma model. Additionally, GFAP mAb reduces pyroptotic cell death in vitro, indicating direct cytoprotective effects. These findings highlight GFAP as a promising therapeutic target and support the potential of GFAP mAb therapy as a novel immunomodulatory approach for glaucoma and other neurodegenerative diseases.

## Electronic supplementary material

Below is the link to the electronic supplementary material.

Supplementary Material 1

Supplementary Material 2

Supplementary Material 3

## Data Availability

The datasets analysed in the bioinformatic re-analysis during the current study are available in the public database PRIDE repository (PXD005258) (https://www.ebi.ac.uk/pride/archive/projects/PXD005258). The datasets generated or analysed supporting the conclusions of this article are included within the article and its supplementary information files.

## Abbreviations

mAb: Monoclonal Antibody
CoCl: Cobalt Chloride
ERG: Electroretinogram
EVO: Episcleral Vein Occlusion
GSDMD: Gasdermin D
Iba1: Ionized Calcium-Binding Adaptor Molecule 1
IL: Interleukin
IF: Intermediate Filament
IVI: Intravitreal Injection
NF-κB: Nuclear Factor Kappa B
NLRP3: NLR Family Pyrin Domain-Containing 3
OCT: Optical Coherence Tomography
RNFL: Retinal Nerve Fiber Layer
ANXA1: Annexin A1
CASP1: Caspase-1
EEF2: Eukaryotic Elongation Factor 2
GFAP: Glial Fibrillary Acidic Protein
HSP90B1: Heat Shock Protein 90 Beta Family Member 1
MAPK: Mitogen-Activated Protein Kinase
MMP9: Matrix Metalloproteinase 9
S100A8: S100 Calcium-Binding Protein A8
S100B: S100 Calcium-Binding Protein B
TLR4: Toll-Like Receptor 4
VIM: Vimentin
TNF-α: Tumor Necrosis Factor-alpha
MMP9: Matrix Metalloproteinase-9
IFN-γ: Interferon-gamma
