# Supplementary material for: Targeting glial fibrillary acidic protein in glaucoma: a monoclonal antibody approach to modulate glial reactivity and neuroinflammation for neuroprotection
Source: J Neuroinflammation. 2025 Jun 17;22:159. doi: 10.1186/s12974-025-03482-8 (PMC12175471; doi:10.1186/s12974-025-03482-8)
Supplement: Supplementary file 1 — Supplementary Material 1 [file 12974_2025_3482_MOESM1_ESM.pdf]

Raw image of western blot

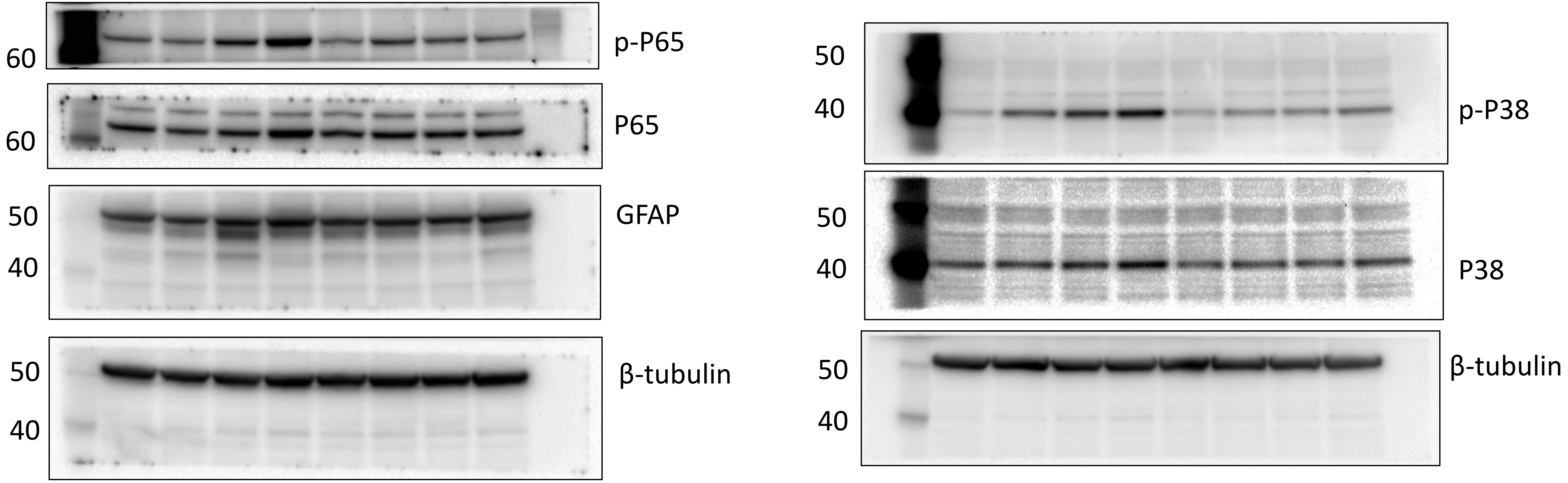

The raw images of Western blot presented in Figure 6 of the manuscript.

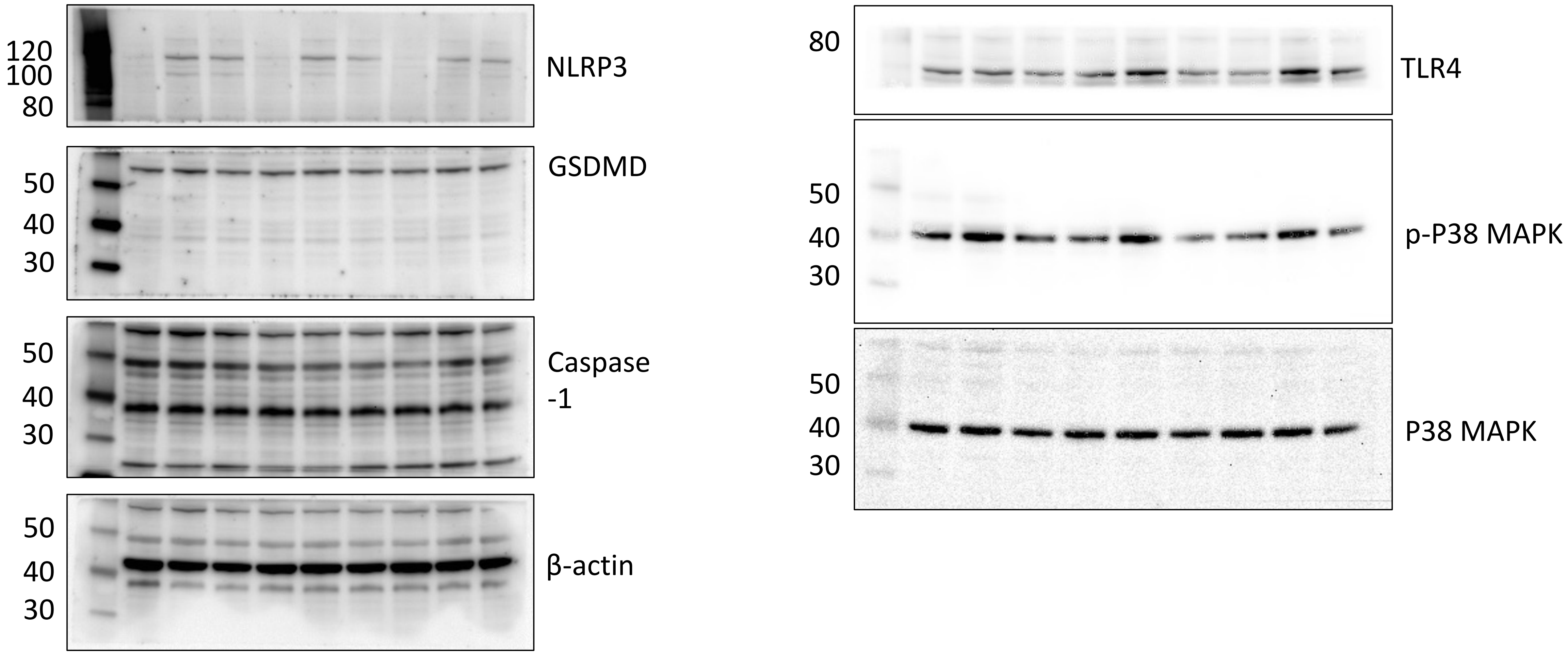

The raw images of Western blot presented in Figure 9 of the manuscript.
