# Supplementary figures and images for: Targeting glial fibrillary acidic protein in glaucoma: a monoclonal antibody approach to modulate glial reactivity and neuroinflammation for neuroprotection
Source: J Neuroinflammation. 2025 Jun 17;22:159. doi: 10.1186/s12974-025-03482-8 (PMC12175471; doi:10.1186/s12974-025-03482-8)

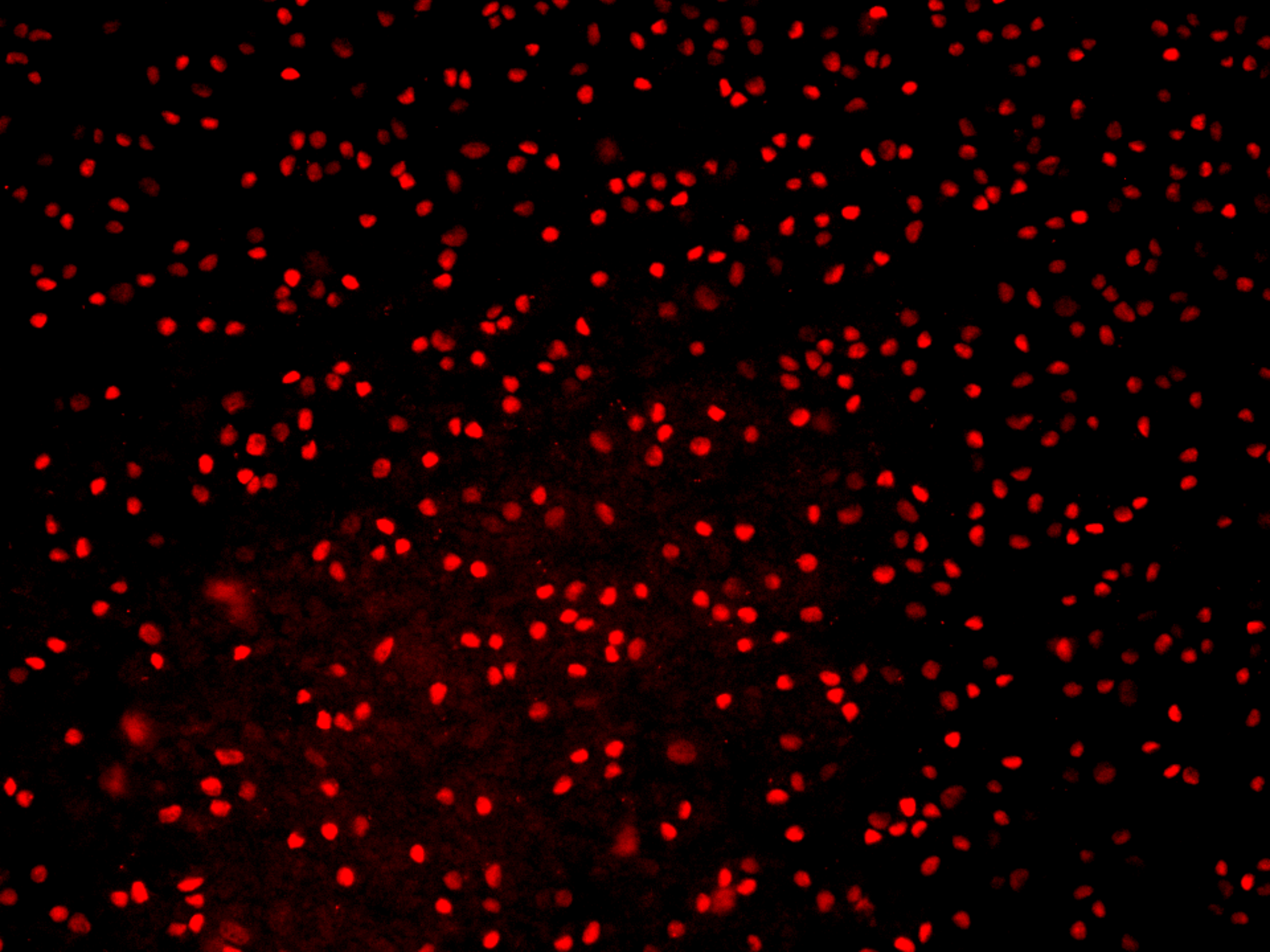

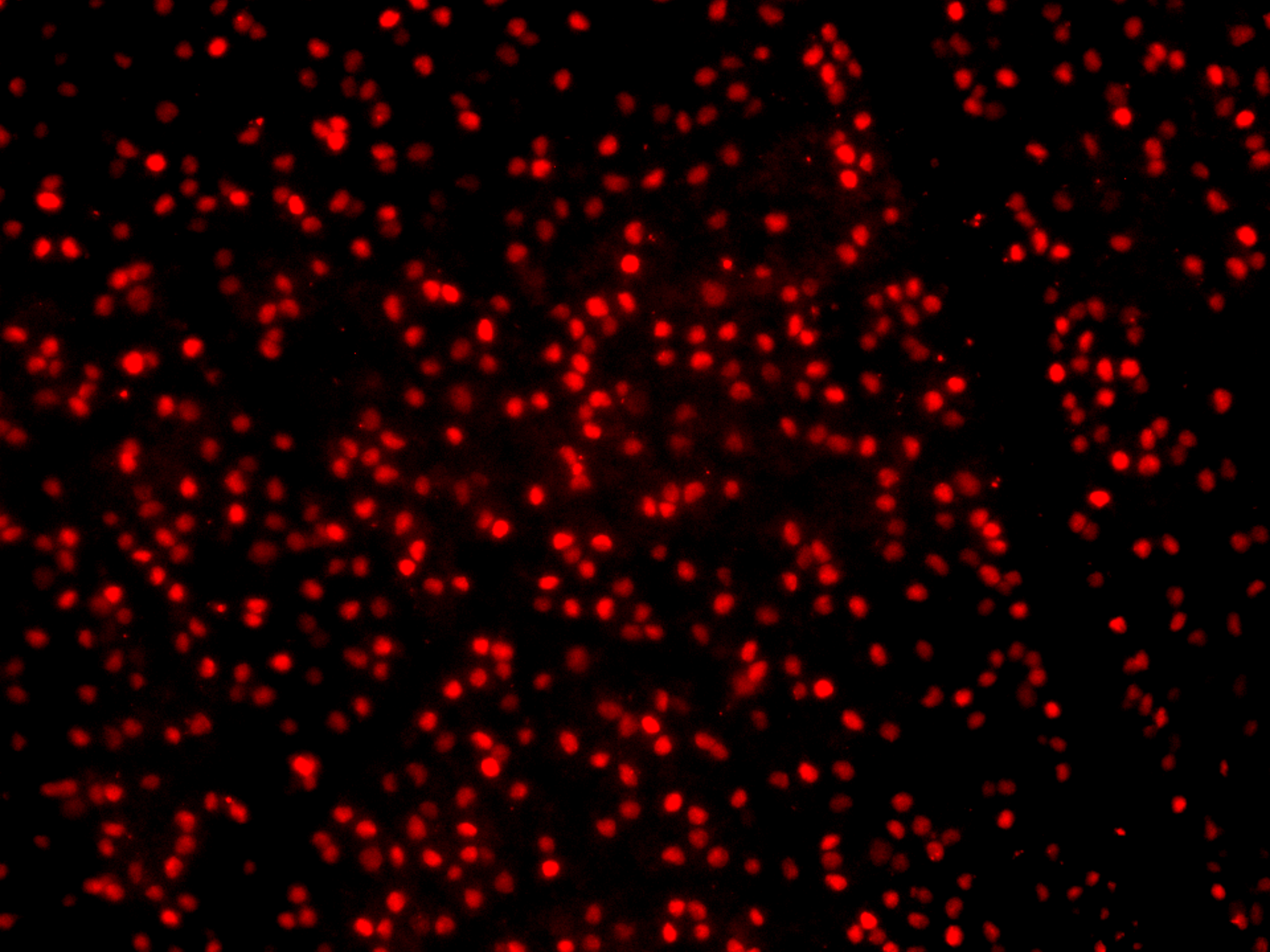

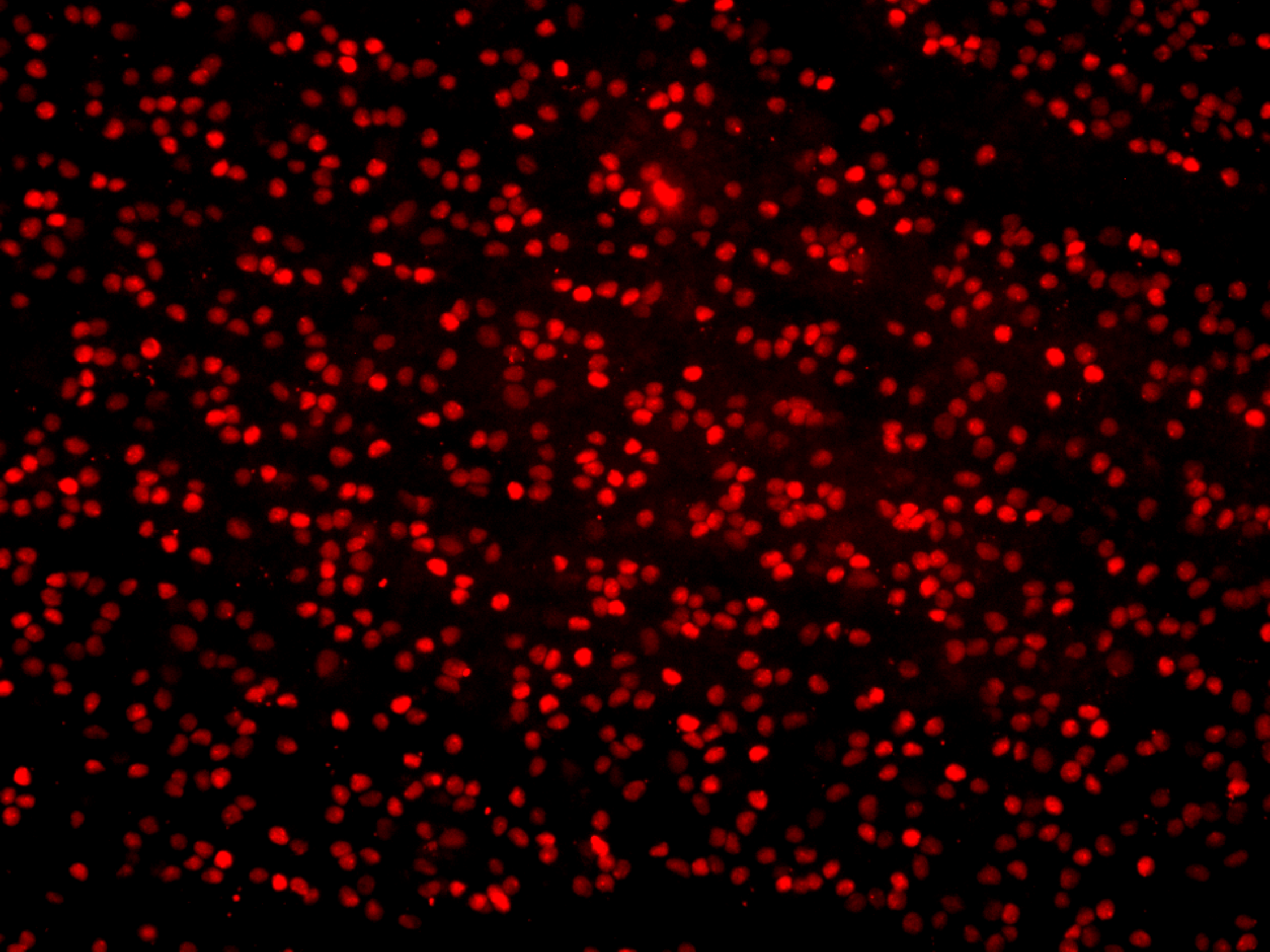

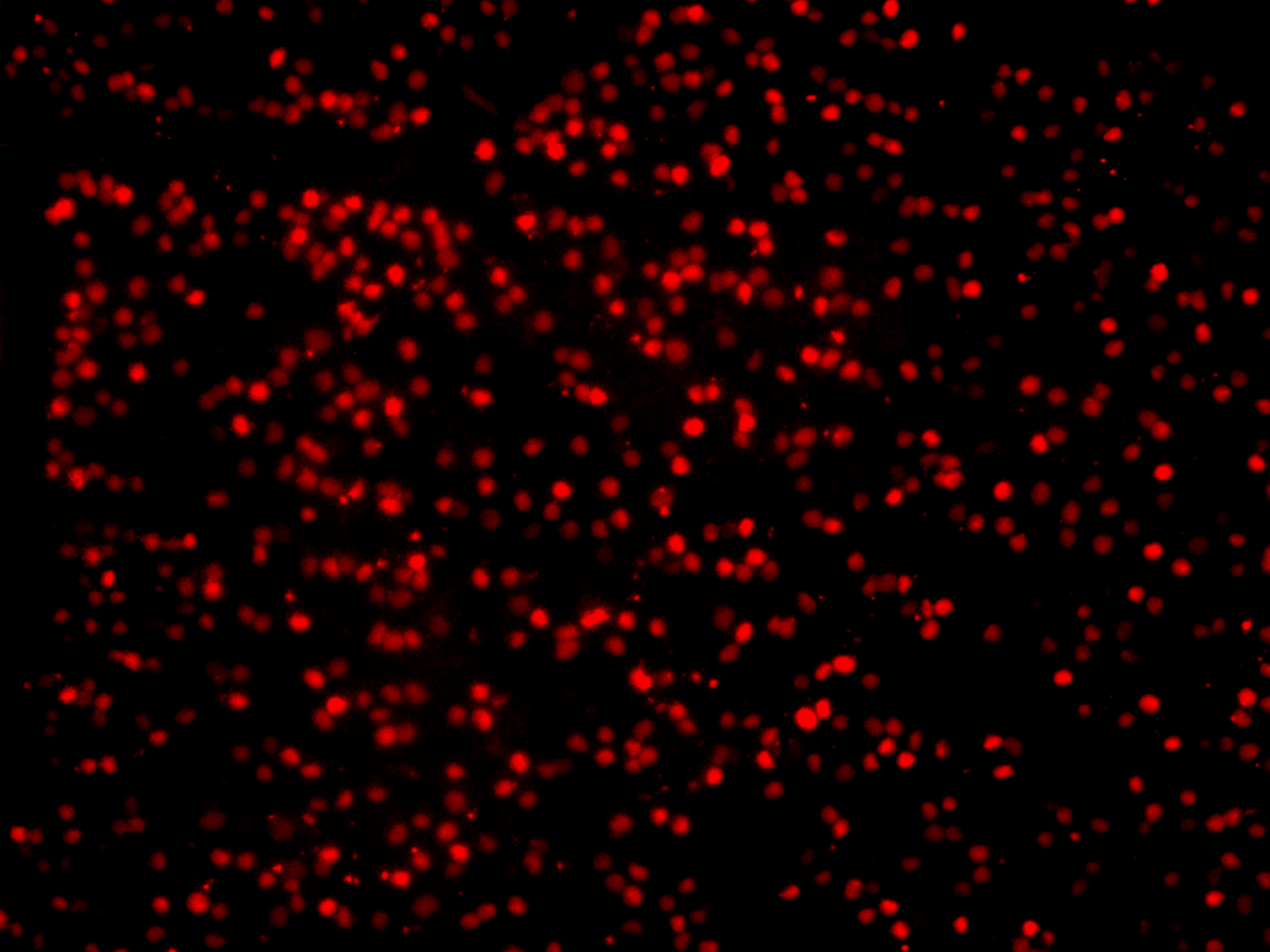

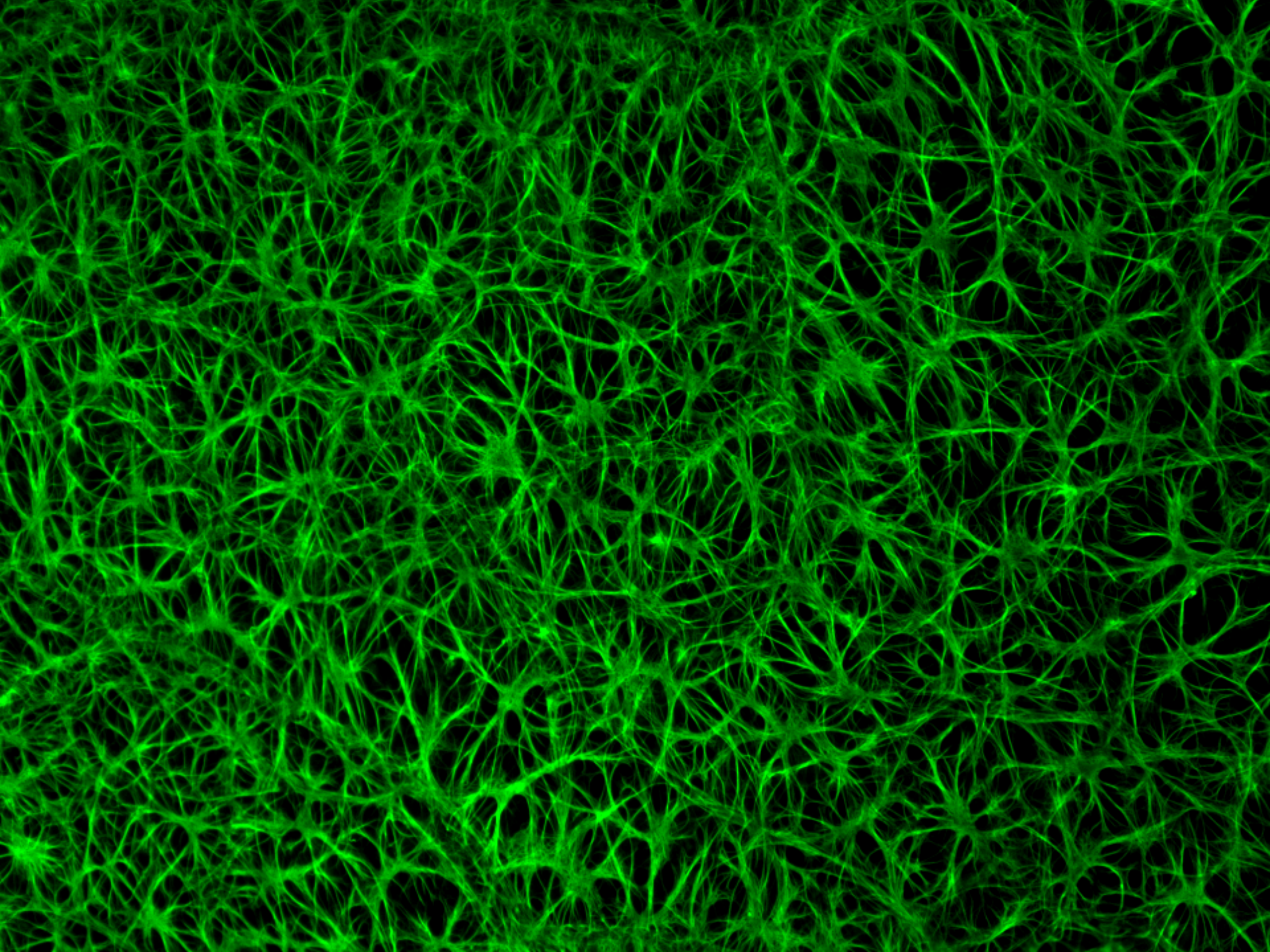

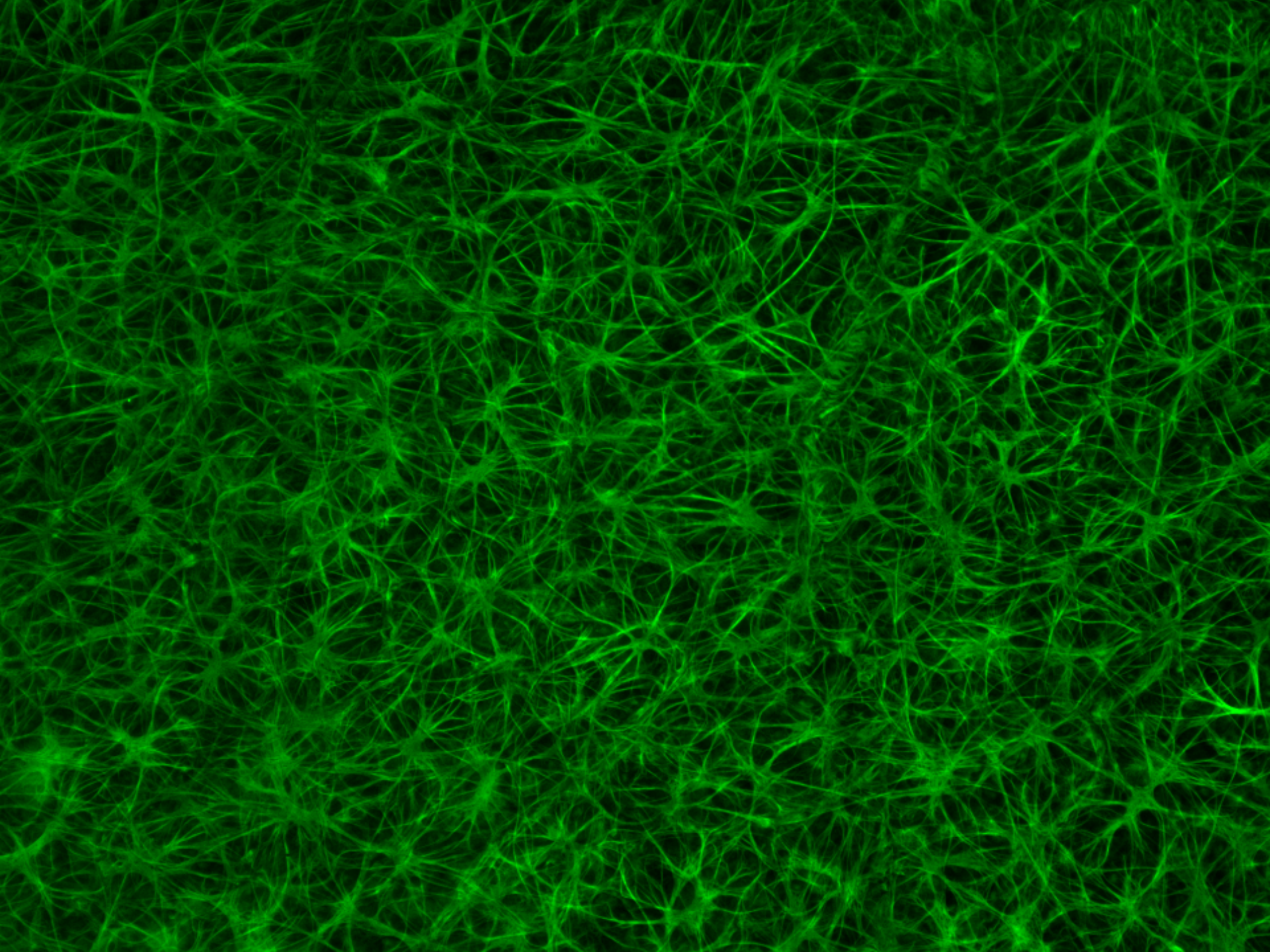

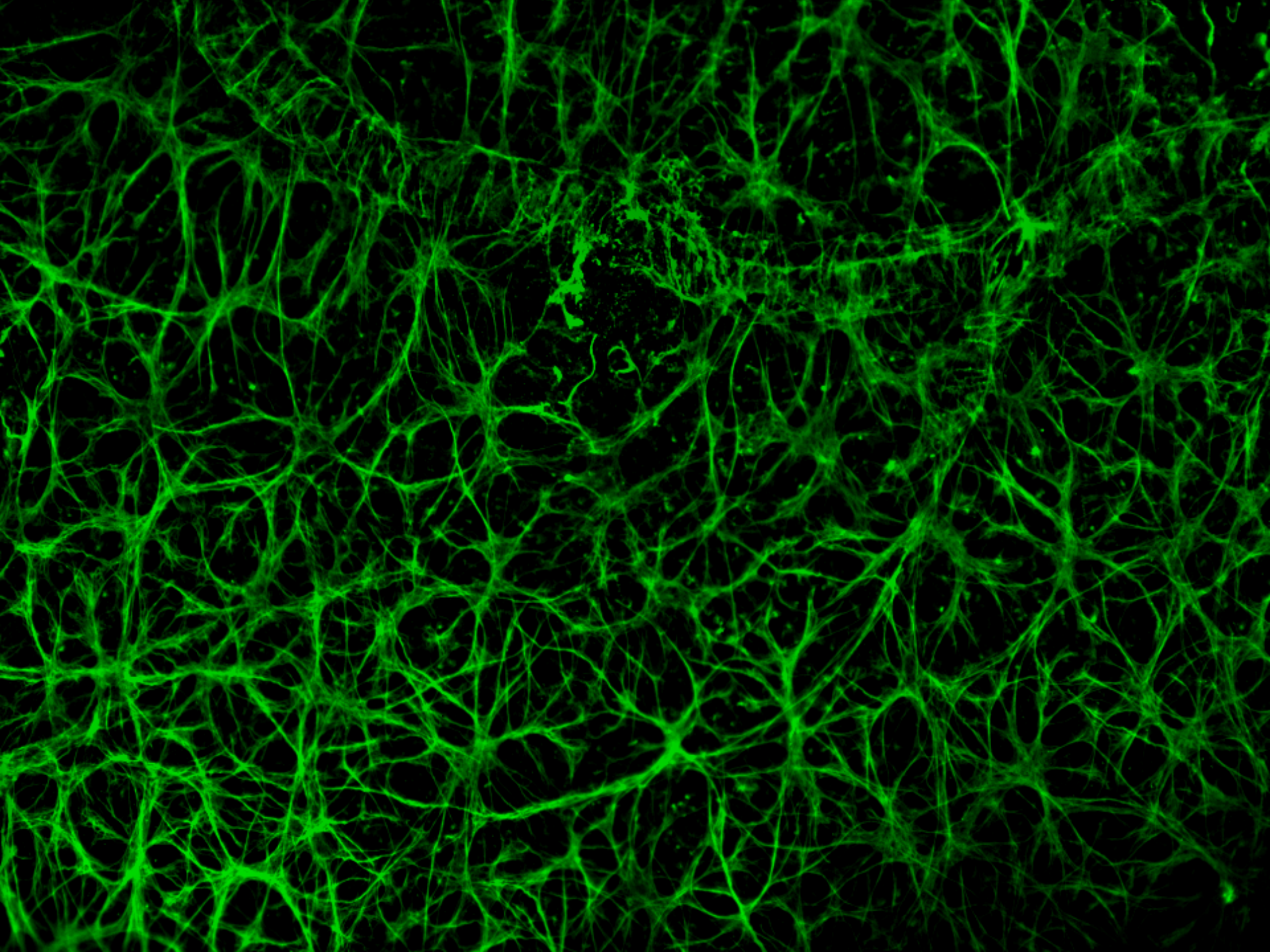

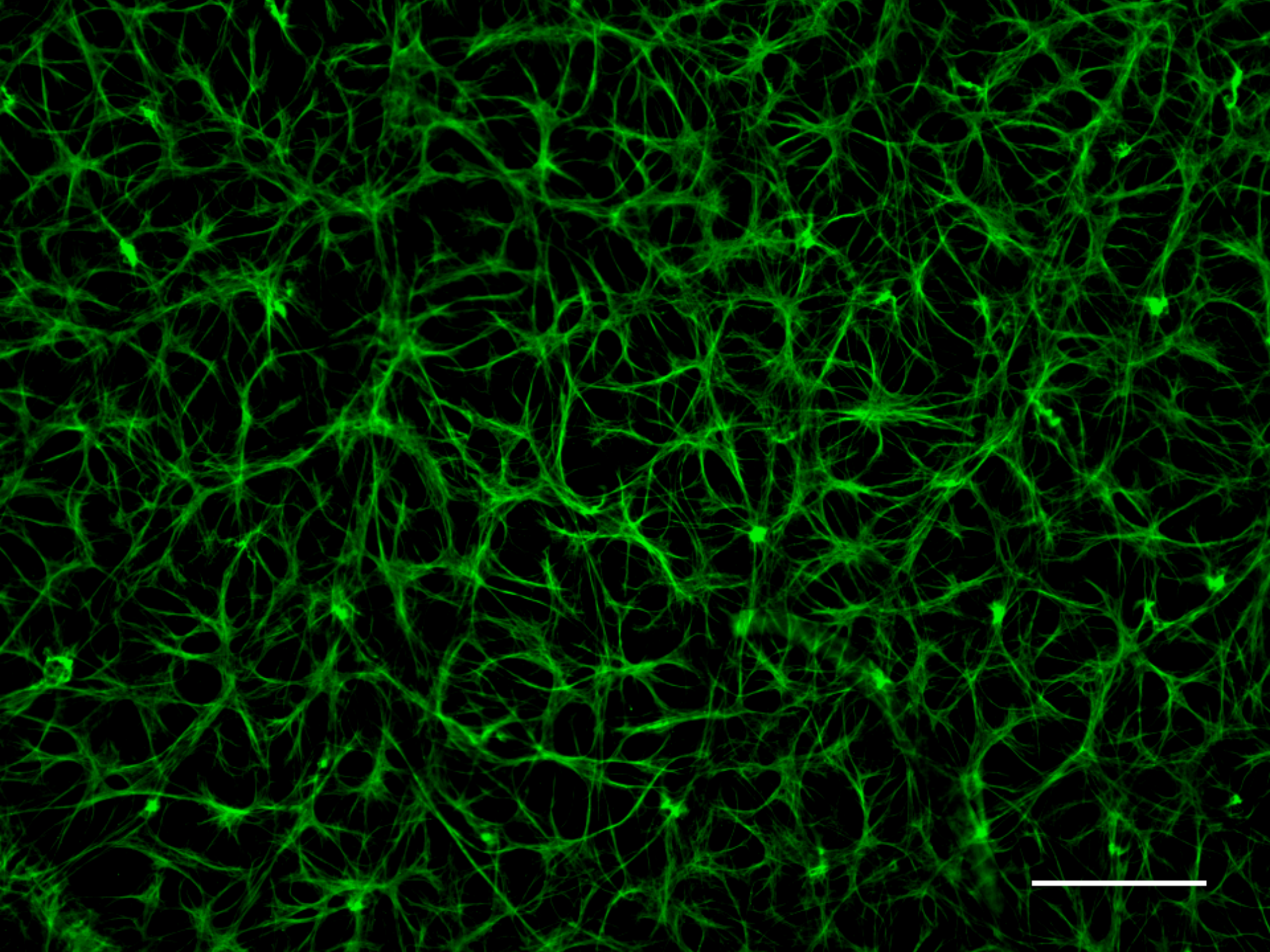

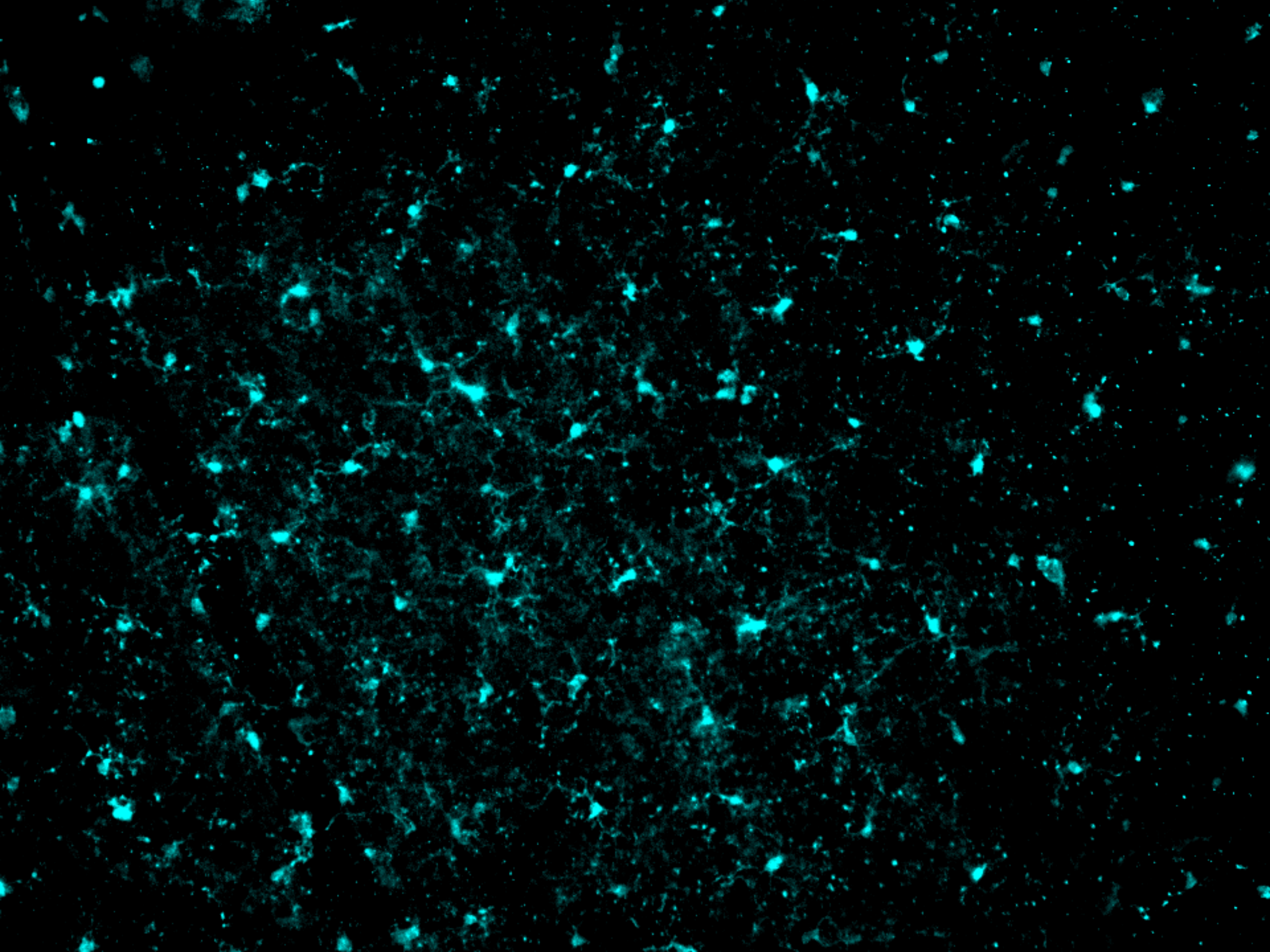

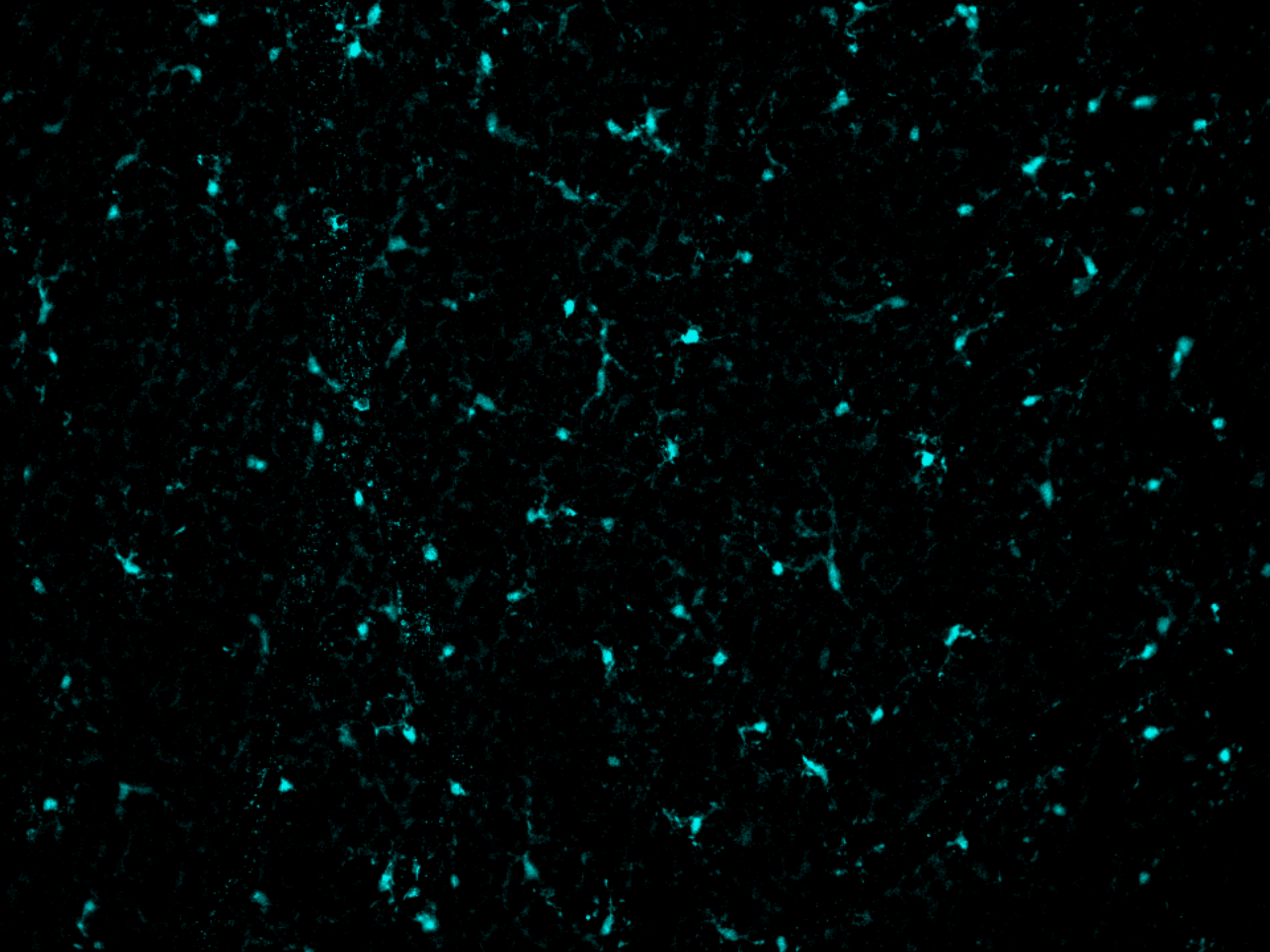

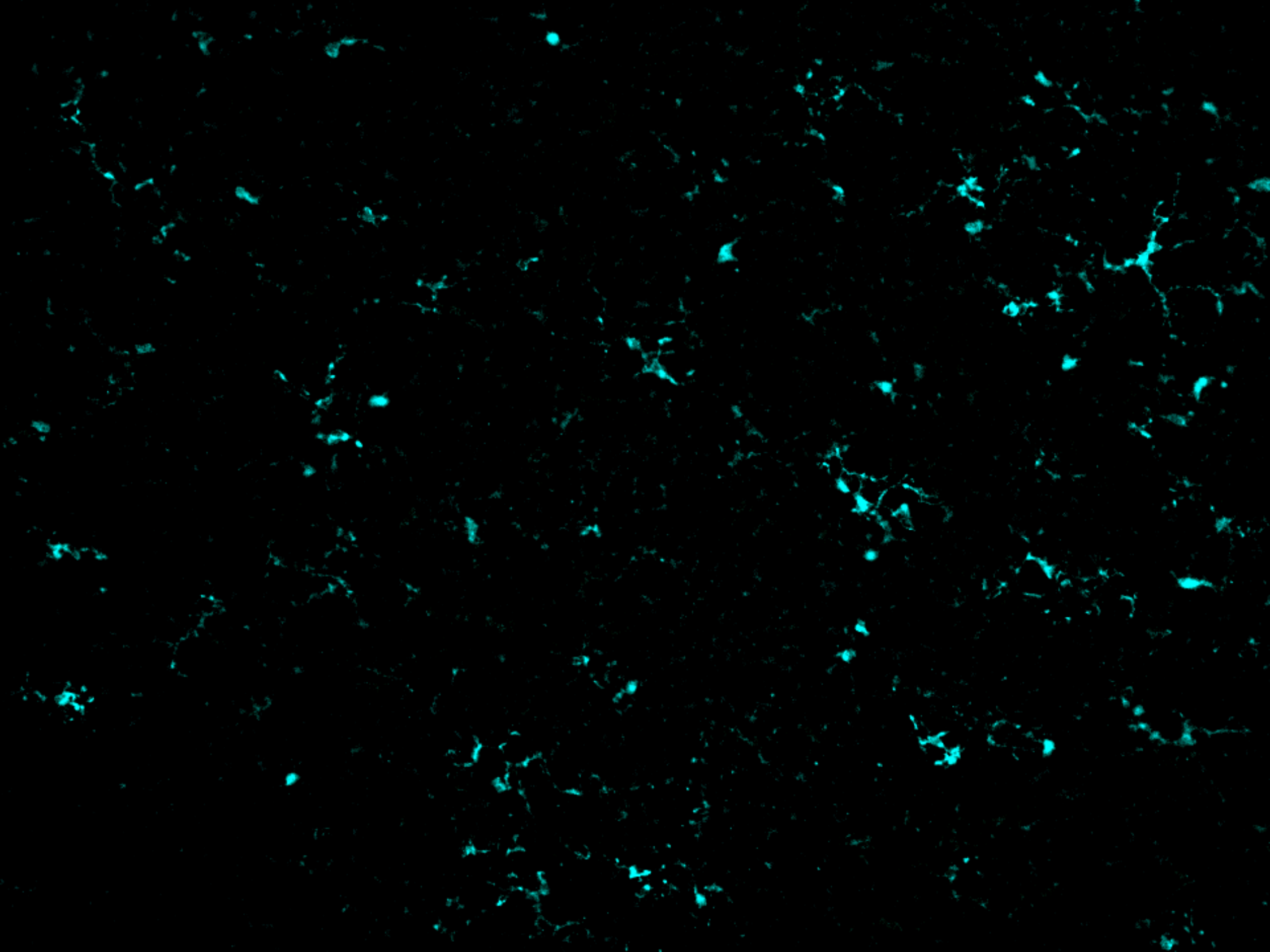

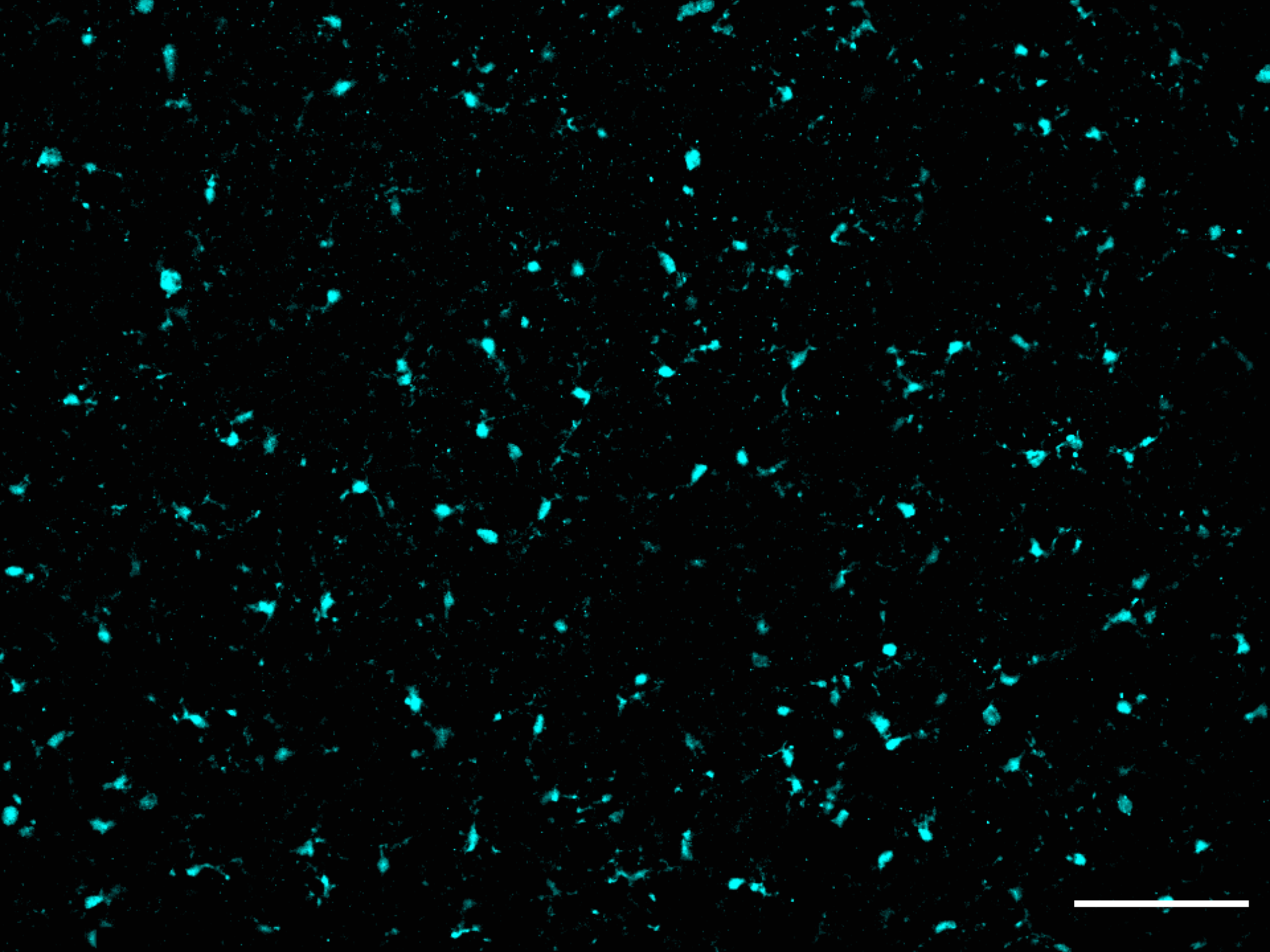

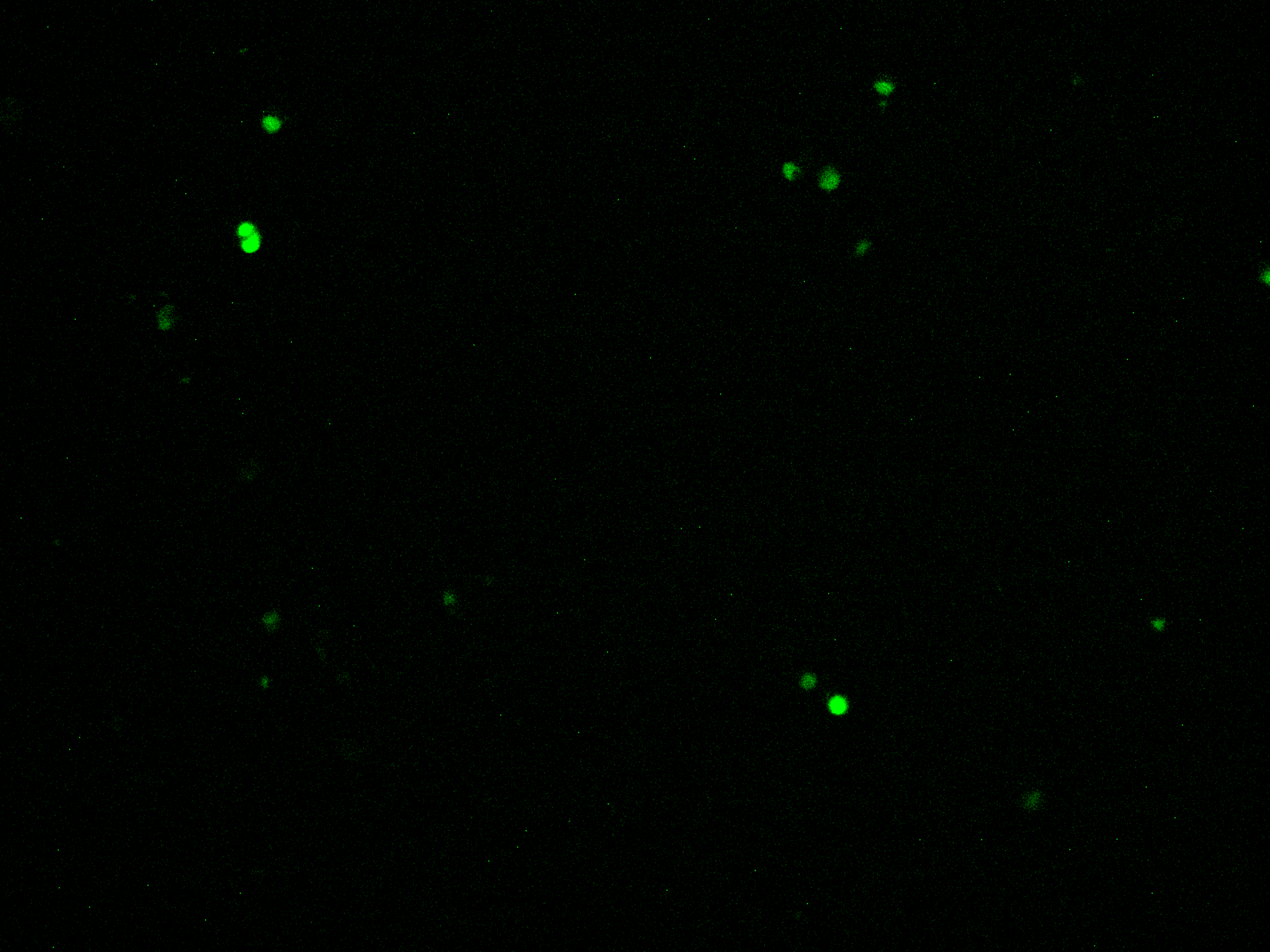



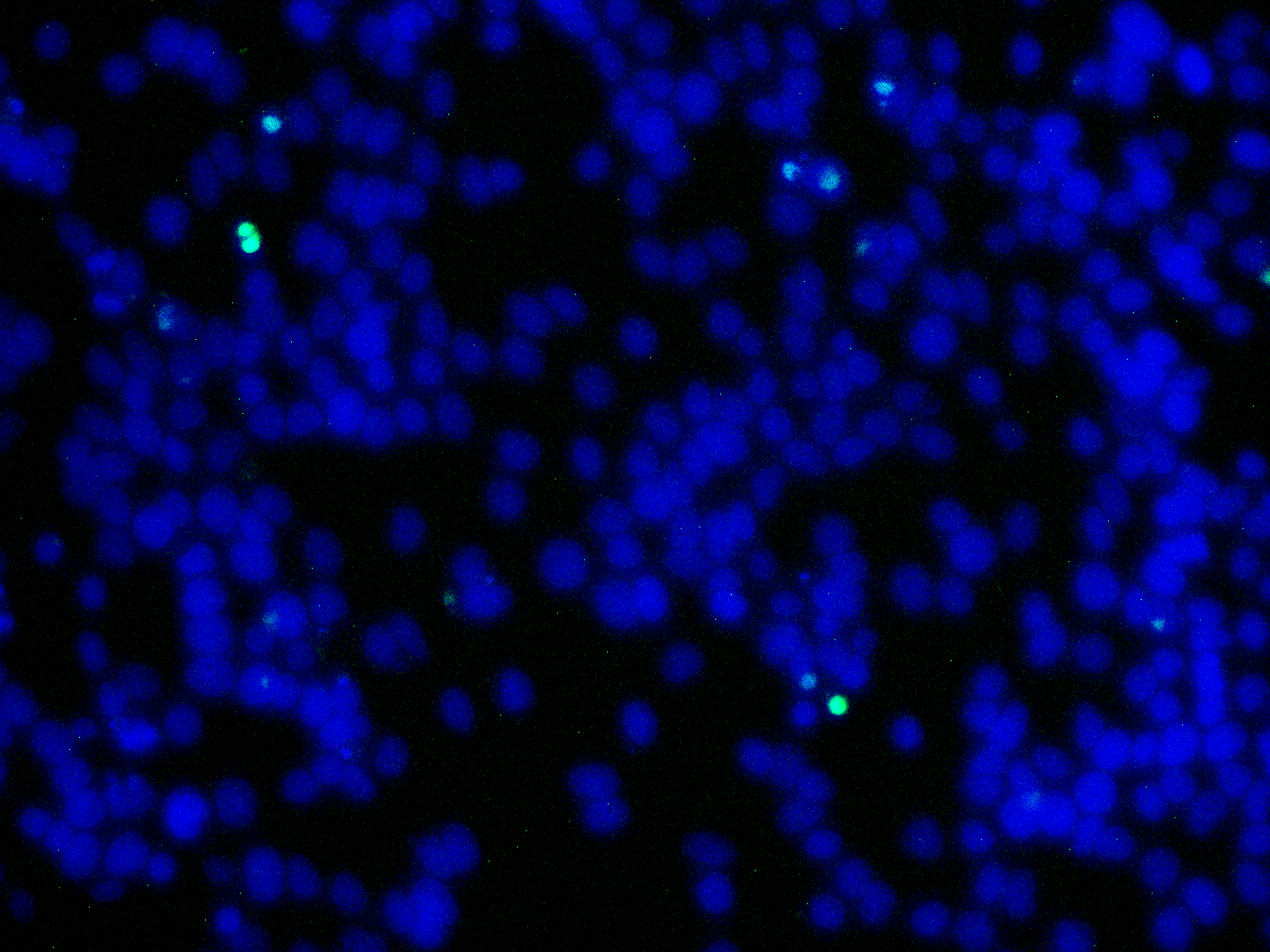

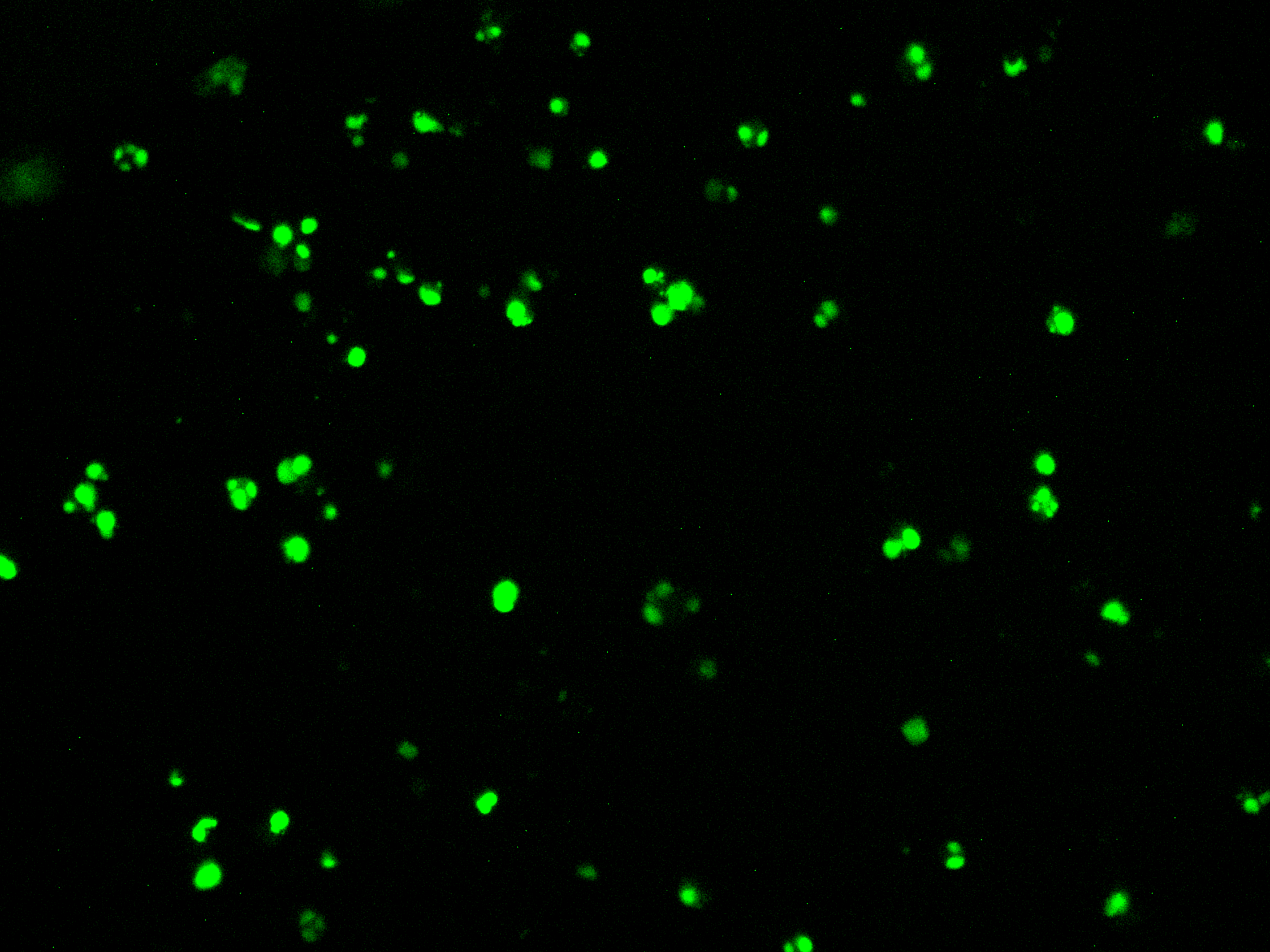



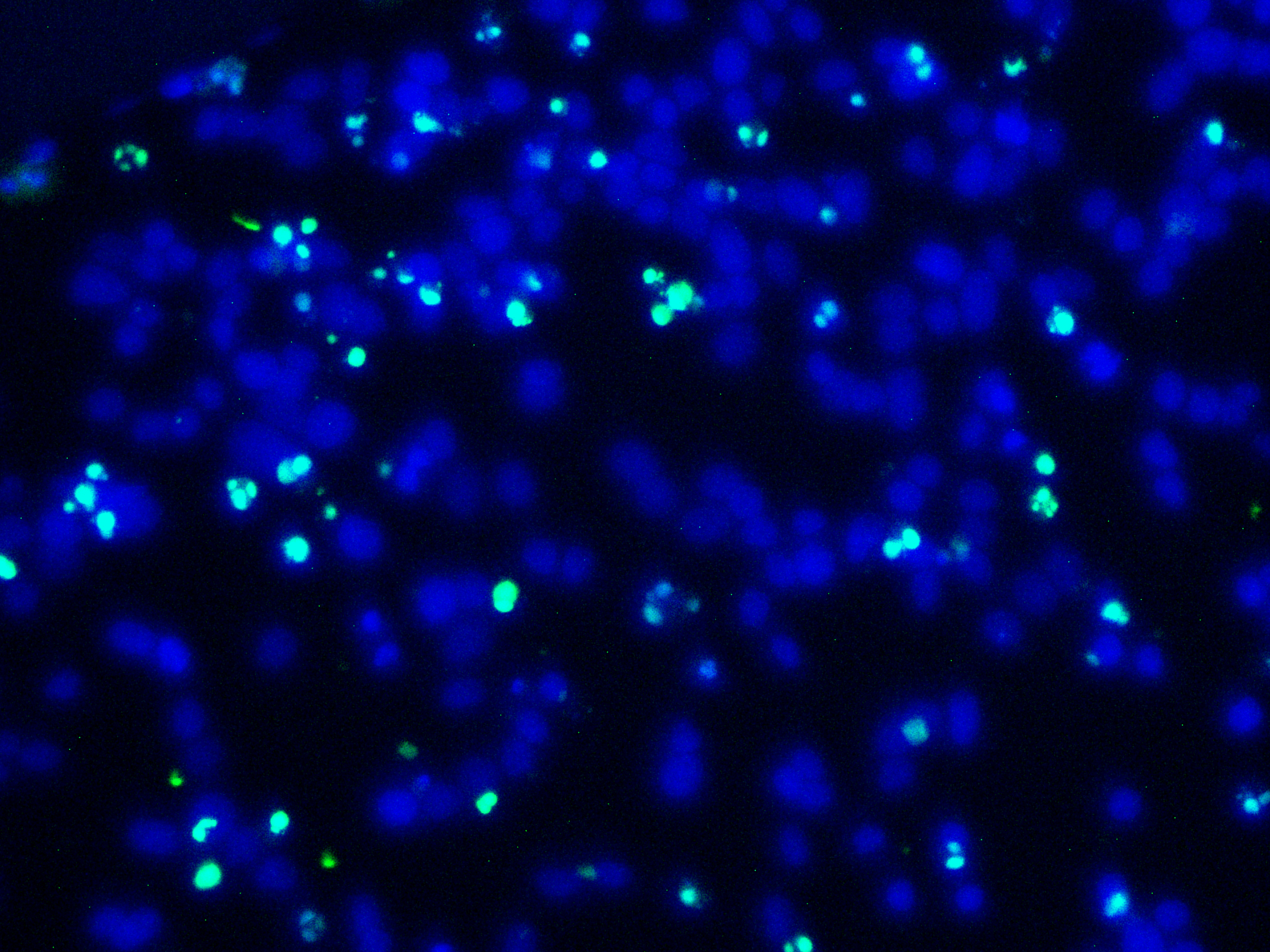

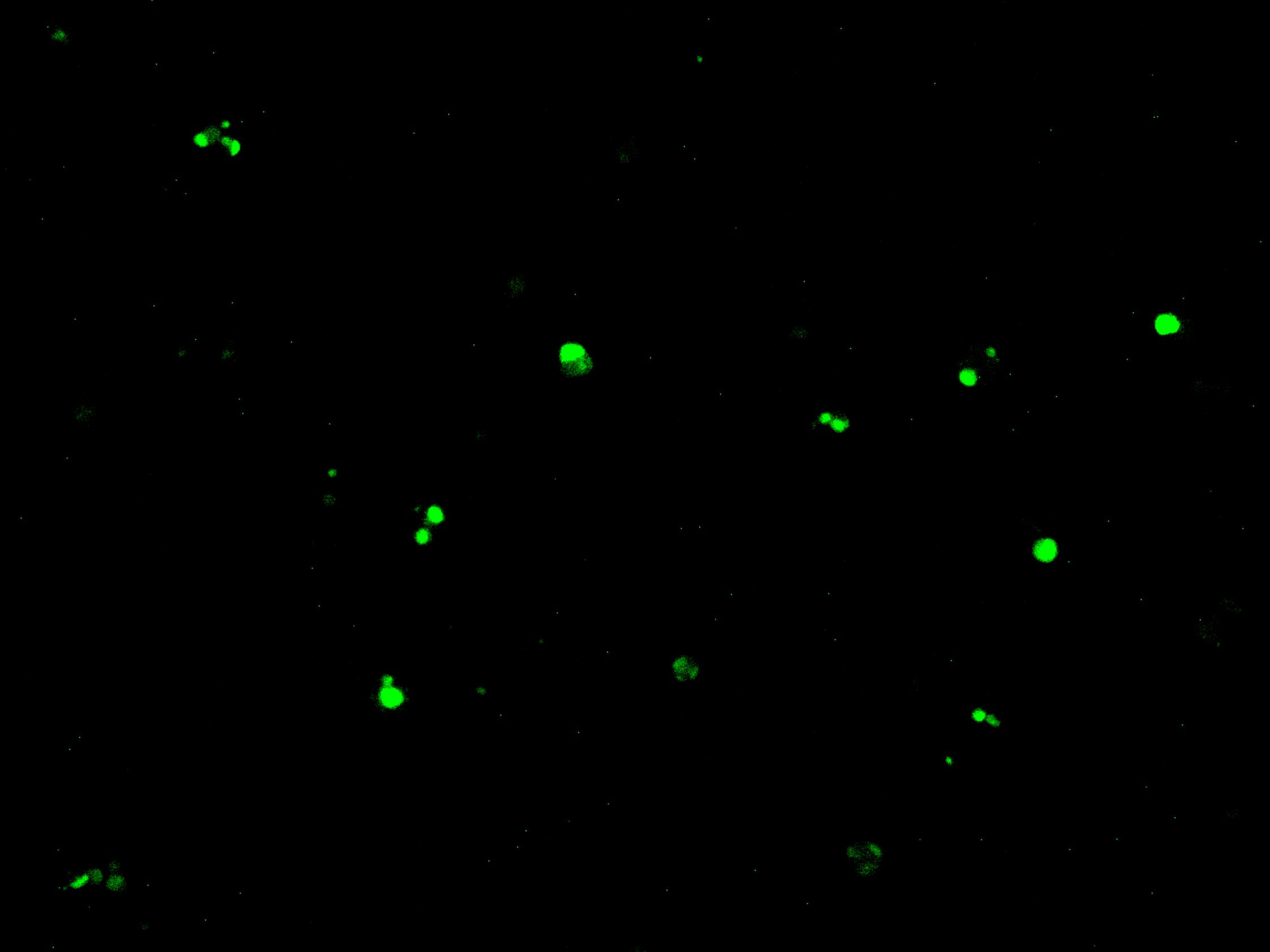



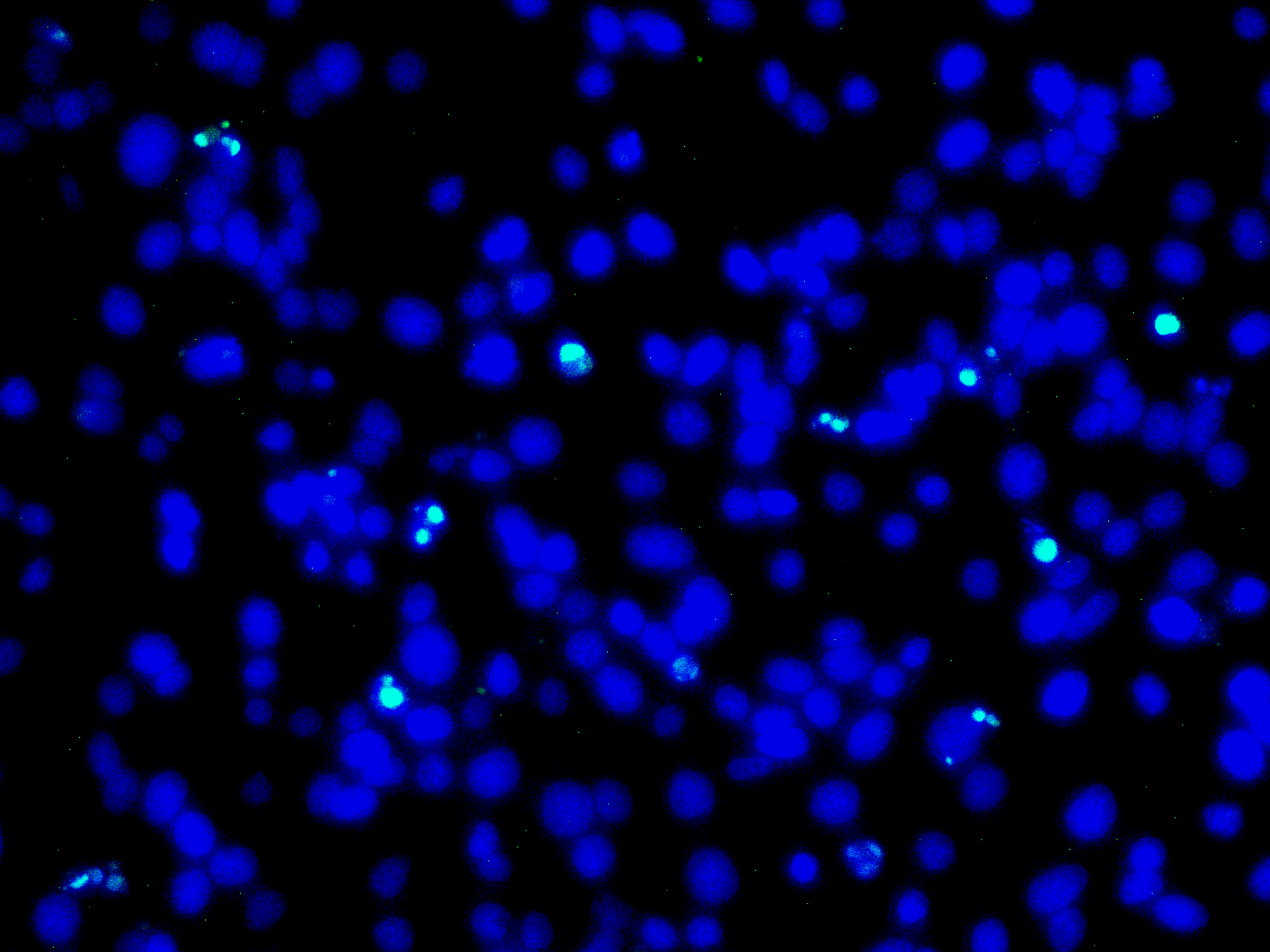

Supplement: Supplementary file 2 — Supplementary Material 2 [file 12974_2025_3482_MOESM2_ESM.pdf]
