# Supplementary material for: Targeting glial fibrillary acidic protein in glaucoma: a monoclonal antibody approach to modulate glial reactivity and neuroinflammation for neuroprotection
Source: J Neuroinflammation. 2025 Jun 17;22:159. doi: 10.1186/s12974-025-03482-8 (PMC12175471; doi:10.1186/s12974-025-03482-8)
Supplement: Supplementary file 3 — Supplementary Material 3 [file 12974_2025_3482_MOESM3_ESM.docx]

| Antibody | Host | Dilution | Catalog | RRID | Manufactor |  |
| --- | --- | --- | --- | --- | --- | --- |
| GFAP monoclonal antibody | Mouse | 1:2000 | 14-9892-80 | AB_1074611 | Invitrogen |  |
| NF-κB p65 monoclonal antibody | Mouse | 1:1000 | 6956 | AB_10828935 | Cell Signaling Technology |  |
| Phospho-NF-κB monoclonal antibody | Rabbit | 1:1000 | 3033 | AB_331284 | Cell Signaling Technology |  |
| p38 MAPK polyclonal antibody | Rabbit | 1:1000 | A14401 | AB_2761271 | Abclonal |  |
| Phospho-p38 MAPK polyclonal antibody | Rabbit | 1:1000 | AP0526 | AB_2771309 | Abclonal |  |
| TLR4 polyclonal antibody | Rabbit | 1:1000 | A14637 | AB_2716293 | Antibodies.com |  |
| NLRP3 polyclonal Antibody | Rabbit | 1:1000 | PA5-121873 | AB_2915445 | Invitrogen |  |
| GSDMD monoclonal antibody | Rabbit | 1:1000 | 39754 | AB_2916333 | Cell Signaling Technology |  |
| caspase-1 polyclonal antibody | Rabbit | 1:1000 | 22915-1-AP | AB_2876874 | Proteintech |  |
| beta-Actin polyclonal antibody | Rabbit | 1:2000 | BS-0061R | AB_10855480 | Bioss |  |
| beta Tubulin monoclonal antibody | Mouse | 1:2000 | 32-2600 | AB_86547 | Invitrogen |  |
| Anti-rabbit IgG H&L (HRP) | Goat | 1:10000 | ab6721 | AB_955447 | Abcam |  |
| Anti-mouse IgG H&L (HRP) | Rabbit | 1:10000 | ab6728 | AB_955440 | Abcam |  |

**Table 1**. Antibodies applied in Western Blot experiment.

**Table 2**. Antibodies applied in Microarray experiment.

| Antibody | Host | Catalog | RRID | Manufactor |
| --- | --- | --- | --- | --- |
| TLR4 polyclonal antibody | Rabbit | A14637 | AB_2716293 | Antibodies.com |
| GSDMD monoclonal antibody | Rabbit | 39754 | AB_2916333 | Cell Signaling Technology |
| Caspase 1 polyclonal antibody | Rabbit | 22915-1-AP | AB_2876874 | Proteintech |
| CD68 polyclonal Antibody | Rabbit | 600-401-MP2 | AB_2614528 | Rockland |
| NLRP3 polyclonal Antibody | Rabbit | PA5-121873 | AB_2915445 | Invitrogen |
| IFN gamma polyclonal antibody | Rabbit | 500-P119-50UG | AB_2929311 | Invitrogen |
| IL-10 polyclonal antibody | Rabbit | 500-P60-50UG | AB_2930649 | Invitrogen |
| IL-6 monoclonal antibody | Mouse | M620 | AB_223576 | Invitrogen |
| IL-8 monoclonal antibody | Mouse | AHC0789 | AB_2536261 | Invitrogen |
| MMP9 polyclonal antibody | Rabbit | ab38898 | AB_776512 | Abcam |
| TNF alpha polyclonal antibody | Rabbit | ab66579 | AB_1310759 | Abcam |
| IL-1 beta polyclonal antibody | Rabbit | ab2105 | AB_302842 | Abcam |
| S100A8 monoclonal antibody | Rabbit | ab92331 | AB_2050283 | Abcam |


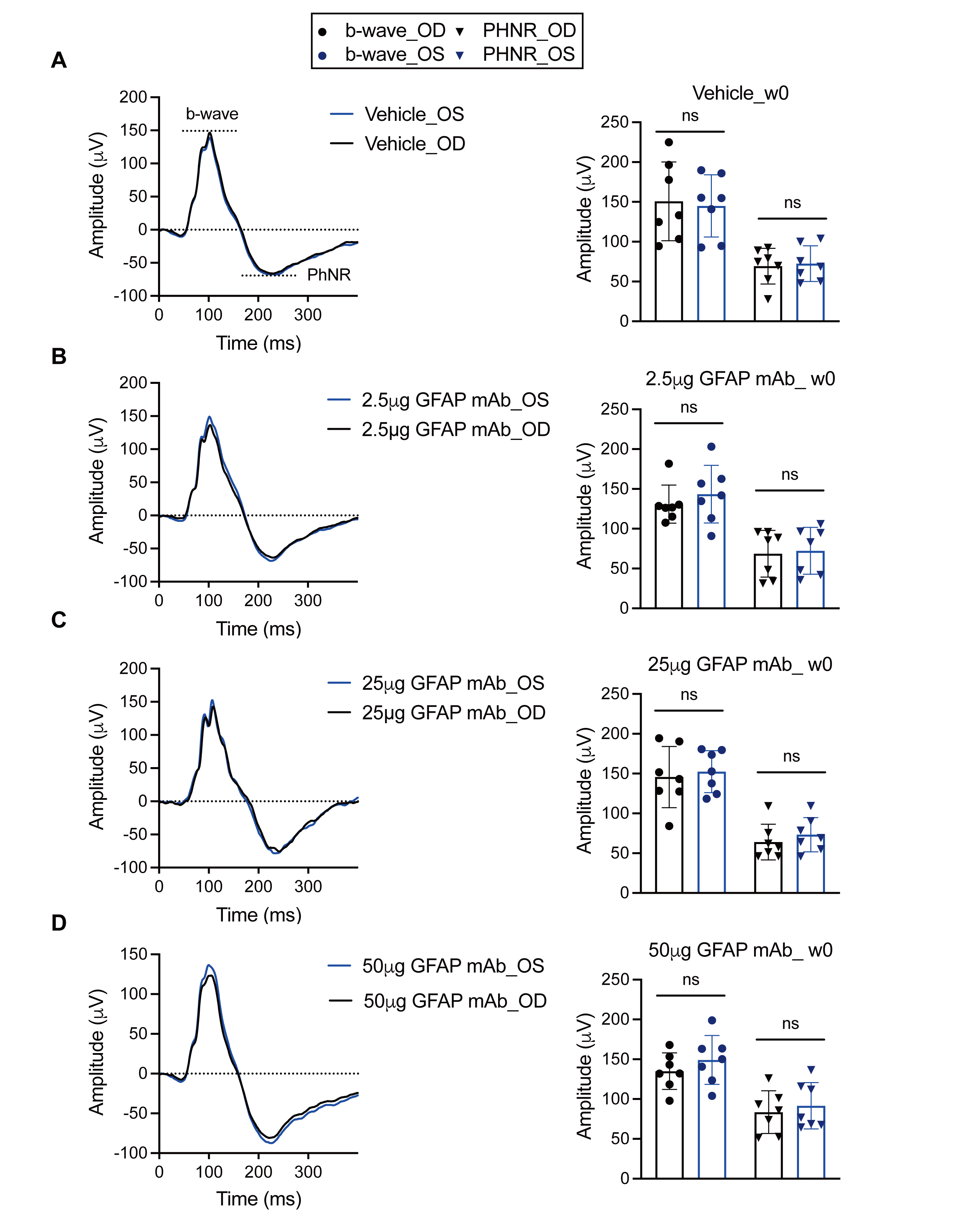


**Figure 1**. Ganzfeld ERG was performed at week 0 (baseline) and the ERG pattern stimulated using a flash intensity of 1.37 log10 cd·s·m^−2^ with the corresponding quantification of b-wave and PhNR amplitudes are shown. (**A**-**D**) Representative ERG recordings from OS and OD in each group at baseline and the corresponding quantification of b-wave and PhNR amplitudes. The results showed that both b-wave and PhNR amplitudes of the OS were not different significantly compared to those of the OD in all four groups. Statistical analysis was performed using paired t-tests, with Holm-Šidák’s correction applied across groups. Data are presented as the mean ± SD. ns = not significant; n = 7 per group
